# Single-Nucleus Profiling Identifies Accelerated Oligodendrocyte Precursor Cell Senescence in a Mouse Model of Down Syndrome

**DOI:** 10.1523/ENEURO.0147-23.2023

**Published:** 2023-08-14

**Authors:** Bianca Rusu, Bharti Kukreja, Taiyi Wu, Sophie J. Dan, Min Yi Feng, Brian T. Kalish

**Affiliations:** 1 Department of Molecular Genetics, University of Toronto, Toronto, Ontario M5G 1A8, Canada; 2 Program in Neuroscience and Mental Health, SickKids Research Institute, Toronto, Ontario M5G 1L7, Canada; 3 Department of Physiology, University of Toronto, Toronto, Ontario M5G 1A8, Canada; 4 Department of Immunology, University of Toronto, Toronto, Ontario M5G 1A8, Canada; 5 Division of Neonatology, Department of Paediatrics, The Hospital for Sick Children, Toronto, Ontario M5G 1L7, Canada

**Keywords:** Down syndrome, oligodendrocyte precursor cells, senescence, single-cell genomics, Trisomy 21

## Abstract

Down syndrome (DS), the most common genetic cause of intellectual disability, is associated with lifelong cognitive deficits. However, the mechanisms by which triplication of chromosome 21 genes drive neuroinflammation and cognitive dysfunction are poorly understood. Here, using the Ts65Dn mouse model of DS, we performed an integrated single-nucleus ATAC and RNA-sequencing (snATAC-seq and snRNA-seq) analysis of the adult cortex. We identified cell type-specific transcriptional and chromatin-associated changes in the Ts65Dn cortex, including regulators of neuroinflammation, transcription and translation, myelination, and mitochondrial function. We discovered enrichment of a senescence-associated transcriptional signature in Ts65Dn oligodendrocyte (OL) precursor cells (OPCs) and epigenetic changes consistent with a loss of heterochromatin. We found that senescence is restricted to a subset of OPCs concentrated in deep cortical layers. Treatment of Ts65Dn mice with a senescence-reducing flavonoid rescued cortical OPC proliferation, restored microglial homeostasis, and improved contextual fear memory. Together, these findings suggest that cortical OPC senescence may be an important driver of neuropathology in DS.

## Significance Statement

Down syndrome (DS) is the most common genetic cause of intellectual disability worldwide, is characterized by chronic neuroinflammation, and results in a ubiquitous incidence of early-onset neurodegeneration. Here, we conduct single-nucleus multiomic profiling of the mature adult cortex of an established mouse model of DS, and systematically identify key perturbations in pathways critical for neurogenesis, myelination, and neuroinflammation. We discover the enrichment of a senescence- associated gene signature in trisomic cortical oligodendrocyte (OL) precursor cells (OPCs), validate our computational findings using orthogonal approaches, and show that a senescence-reducing flavonoid significantly improves memory deficits. Our findings suggest that OPC senescence may play a role in the pathogenesis of DS and that senescence-reducing treatment may provide a novel approach for improving cognitive dysfunction in DS.

## Introduction

Down syndrome (DS) is the leading genetic cause of intellectual disability worldwide, occurring in one in ∼800 live births (Alexander et al., 2016; Kazemi et al., 2016) and is caused by the complete or partial triplication of *Homo sapiens* chromosome 21 (Hsa21; Antonarakis et al., 2020). DS results in learning, memory, and language impairment, leading to lifelong cognitive disability, as well as a universal risk of early-onset neurodegeneration (Zigman et al., 2008). Triplication of Hsa21 results in increased expression of ∼225 protein coding genes, as well as >300 genes of unknown coding or functional potential (Sturgeon and Gardiner, 2011). Overexpression of these genes results in complex perturbations of multiple processes involved in neurologic development and function (Becker et al., 1991; Barlow et al., 2002; Chakrabarti et al., 2007, 2010; Jose Luis Olmos-Serrano et al., 2016).

DS presents a unique opportunity for studying changes in brain aging across the lifespan, as DS individuals age prematurely and atypically (Antonarakis et al., 2020). Importantly, gene dosage imbalance of this relatively small set of genes has a profound cascading effect, including secondary changes in expression of hundreds of other genes, leading to the disruption of several pathways important for neurogenesis, cell differentiation, synapse formation and plasticity, axon guidance, immune regulation, and myelination (Becker et al., 1991). By 40 years of age, there is a ubiquitous occurrence of plaques and neurofibrillary tangles of hyperphosphorylated tau suggestive of Alzheimer’s disease (AD), as well as clinical signs of dementia, including changes in sociability, language, and depressive symptoms (Teller et al., 1996; Head et al., 2016; Godfrey and Lee, 2018).

Neuroinflammation, a key driver of neurodegeneration, is a neuropathologic hallmark of DS (Wilcock, 2012; Flores-Aguilar et al., 2020) and is in part driven by triplication of several immune-related genes, including four of the six interferon (IFN) receptors (Sullivan et al., 2016; Kong et al., 2020). There is also triplication of the amyloid precursor protein (APP) and S100β, with the resultant overexpression of the pluripotent neuroinflammatory cytokine interleukin-1 (IL-1; Lu et al., 2011; Wilcock and Griffin, 2013; Head et al., 2016). Importantly, there is evidence of microglial activation in DS humans and mice with trisomy, and reducing overactivated microglia restores cognitive performance in trisomic mice (Pinto et al., 2020). While neuroinflammation is recognized as a core feature of DS, the molecular mechanisms and age-related progression of inflammation-associated aging (“inflammaging”), as well as their relationship to cognitive decline, are poorly understood.

To address these gaps, we leveraged single-nucleus sequencing to identify candidate pathways perturbed in the cortex of a well-characterized DS mouse model. Ts65Dn mice contain a partial triplication of *Mus musculus* chromosome 16 (Mmu16) corresponding to trisomy of ∼55% of Hsa21 protein-coding orthologs (Reeves et al., 1995; Hyde et al., 2001; Gupta et al., 2016). These trisomic mice recapitulate many of the characteristics of human DS, including learning, memory, and cognitive deficits, as well as developmental delays and oligodendrocyte (OL) maturation deficits (Granholm et al., 2000; Hyde et al., 2001; Gupta et al., 2016). Through multiomic profiling of the mature Ts65Dn cortex, we identify broad disruptions in pathways associated with neuroinflammation, transcriptional and translational regulation, mitochondrial and ribosomal dysfunction, and the integrated stress response (ISR) at both the epigenomic and transcriptomic level across neuronal and non-neuronal cell populations. We further identify a senescence-associated gene signature in Ts65Dn oligodendrocyte precursor cells (OPCs). Using orthogonal approaches, we validate our sequencing findings and discover that a subset of cortical OPCs are prematurely senescent in the trisomic mouse brain. We show that treatment of Ts65Dn mice with the anti-senescence flavonoid, fisetin, has restorative effects on cortical OPCs, including reducing senescence-associated-β-galactosidase (SA-β-gal) activity, increasing proliferation and progenitor abundance, restoring microglia homeostasis, and improving contextual fear memory. In sum, this work identifies novel cellular mechanisms that drive chronic neuroinflammation and cognitive dysfunction in the Ts65Dn mouse model of DS.

## Materials and Methods

### Mice

All animal experiments were performed in accordance with the Canadian Council of Animal Care policies. Ts65Dn (*B6EiC3Sn.BLiA-Ts(1716)65Dn/DnJ*, RRID:IMSR_JAX:005252) and euploid controls (*B6EiC3Sn.BLiAF1/J*, RRID:IMSR_JAX:003647) were purchased from The Jackson Laboratory. Ts65Dn mice were bred with euploid controls as recommended by The Jackson Laboratory. The animals were kept in 12/12 h light/dark cycles with free access to food and chow, and bred at The Center for Phenogenomics in Toronto (ON, Canada). For all studies, the sex of the mice is listed in the respective figure legend. The specific ages of each animal for each experiment are documented in the respective results, method details, and/or figure legends of the study. A cohort of Ts65Dn mice, designated for the Ts65Dn+fisetin condition, received a custom diet produced by Envigo, containing 500 ppm fisetin (Indofine Chemical, catalog #528-48-3) in the chow, starting at three months of age, until euthanasia at six months. Quantitative PCR (qPCR) was performed on genomic DNA extracted from tail tips of all mice to confirm individual genotype. The primers used are as follows: 11,981 mutant A, GTGGCAAGA GACTCAAAT TCAAC; 11,982 mutant A, TGGCTTATT ATTATCAGG GCATTT; OIMR7338 IC forward, CTAGGCCAC AGAATTGAA AGATCT; OIMR7338 IC reverse, GTAGGTGGA AATTCTAGC ATCATCC.

### Tissue preparation for immunostaining

For immunostaining, Ts65Dn, fisetin-treated (Ts65Dn+fisetin), and euploid control (CTL) mice were perfused intra-aortically with a solution of 4% paraformaldehyde (PFA; Electron Microscopy Sciences, catalog #15710) while under isoflurane anesthesia (for untreated Ts65Dn and CTL groups at three months: *n *=* *4 per condition; for Ts65Dn, Ts65Dn+fisetin, and CTL groups at six months: *n* = 3 per condition). Brains were removed and postfixed in 4% PFA at 4°C overnight, then washed 3× for 20 min in 1× PBS before cryoprotection in a 30% sucrose solution at 4°C for 3–4 d. The tissue was embedded in optimal cutting temperature (O.C.T.) compound (Fisher Scientific, catalog #23-730-571) and allowed to solidify at −80°C. Brains were then sliced coronally at 20 μm on a cryostat, mounted onto 0.5% gelatin-coated superfrost slides (Fisher Scientific, catalog #22-037-246) and stored at −80°C long term; three sequential sections were collected per slide.

### Senescence-associated-β-galactosidase (SA-β-gal) staining

SA-β-gal activity was determined on coronal brain sections using a SA-β-gal kit (Cell Signaling, catalog #9860) according to the manufacturer’s instructions. Briefly, sections were incubated at 37°C for 20 min, washed 3× in 1× PBS for 10 min each, fixed for 10 min at room temperature using the kit’s fixative agent, and stained overnight for 16 h using the SA-β-gal staining solution (pH 5.9–6.1, prepared according to kit instructions) in a sealed container placed in a CO_2_-free incubator. The sections were then washed in 1× PBS for 10 min and double-distilled water (DDW) for 5 min before coverslip-mounting with mounting medium (ThermoFisher, catalog #TA030FM).

### Immunohistochemistry (IHC)

When coupled with SA-β-gal staining, the above protocol was followed with several additional steps. After washing in 1× PBS and deionized water, slides were washed in TBS (0.1 m Tris-HCl pH 7.5, 0.15 m NaCl in DDW) for 10 min, permeabilized with TBS-Tx (0.4% Triton X-100 in TBS) 2× for 10 min each, and blocked in blocking buffer (5% BSA, 0.4% Triton X-100 in TBS) at room temperature for 1 h. The primary antibody was diluted in blocking buffer and applied to the slide, incubated in a humidified chamber overnight at 4°C. Staining was subsequently performed using HRP/DAB Detection kits (Abcam, catalog #Ab64261 or Ab64259) according to the manufacturer’s instructions, with minor adaptations. Briefly, sections were washed 4× in PBS-T (0.2% Triton X-100 in 1× PBS), incubated with biotinylated goat anti-polyvalent for 10 min, washed 4× in PBS-T, incubated with streptavidin peroxidase for 10 min, washed 4× in PBS-T, incubated with DAB mixture (2% DAB chromogen in DAB substrate) for 8–10 min, washed 4× in 1× PBS, and mounted with mounting medium (ThermoFisher, catalog #TA030FM).

### Immunofluorescence (IF)

Sections were incubated at 37°C for 20 min, washed 3× in 1× PBS for 10 min each, and blocked in blocking buffer (10% BSA, 6% NDS, 0.3% Triton X-100 in 1× PBS) at room temperature for 1 h. The primary antibody was diluted in blocking buffer and applied to the slide, incubated in a humidified chamber overnight at 4°C. Sections were subsequently washed 3× in PBS-T (0.1% Triton X-100 in 1× PBS) for 10 min, and incubated with secondary antibody diluted in PBS-T for 1 h at room temperature. After incubation, the sections were washed in 1× PBS and mounted with Fluoromount mounting medium (ThermoFisher, catalog #00-4959-52).

### Antigen retrieval (AR)

When necessary, AR was coupled with the above IF protocol following the first round of 3 × 10 min 1× PBS washes. Sodium citrate buffer (10 mm sodium citrate, 0.05% Tween 20, pH 6.0) was created according to Abcam’s protocol for heat-induced epitope retrieval. Slides were completely submerged in the buffer and heated for 20 min at full power in a domestic microwave once the solution reached boiling point. When 20 min elapsed, the heating vessel was removed and cooled in a bath of cold water until it reached room temperature. Slides were then washed 3× in 1× PBS for 10 min each and the aforementioned IF protocol was resumed beginning with 1 h incubation in blocking buffer.

### Antibody concentrations

The primary antibodies used for immunostaining were goat anti-PDGFRA (1:250, R&D Systems, catalog #AF1062), rabbit anti-PDGFRA (1:250, Abcam, catalog #Ab203491), rabbit anti-OLIG2 (1:500, Abcam, catalog #Ab136253), mouse anti-CC1 (1:100, CalBioChem, catalog #OP80), rabbit anti-IBA1 (1:500, Wako, catalog #019-19741), rabbit anti-LMNB1 (1:1000, Abcam; used with AR, catalog #Ab16048), mouse anti-NEUN (1:500, Millipore, catalog #Ab377), mouse anti-AQP4 (1:100, Abcam, catalog #Ab9512), rabbit anti-KI67 (1:200, Abcam, catalog #Ab16667), rat anti-CD68 (1:400, Abcam, catalog #Ab53444), rabbit anti-MBP (1:250, Abcam, catalog #Ab40390). The secondary antibodies used for immunostaining were Alexa Fluor 488 goat anti-rabbit IgG (1:500, ThermoFisher, catalog #A-11034), Alexa Fluor 555 goat anti-mouse IgG (1:500, ThermoFisher, catalog #A-21422), Alexa Fluor 555 goat anti-rat IgG (1:500, ThermoFisher, catalog #A-21434), Alexa Fluor 488 donkey anti-goat (1:500, ThermoFisher, catalog #A-11055), Alexa Fluor 555 donkey anti-mouse IgG (1:500, ThermoFisher, catalog #A-31570), and Alexa Fluor 647 donkey anti-rabbit (1:500, ThermoFisher, catalog #A-31573).

### Quantitative PCR (qPCR) analysis

mRNA expression levels of *Mbp* were measured by qPCR analysis. qPCR was performed on six-month tissue for Ts65Dn, Ts65Dn+fisetin, and CTL groups (*n *=* *4 per condition). In brief, cortical tissue was lysed in 1 ml of TRIzol (ThermoFisher, catalog #15596026) and total RNA was isolated according to the manufacturer’s guidelines. cDNA synthesis was performed using 2 μg of total RNA and the iScript cRNA synthesis kit (Bio-Rad, catalog #1708890). qPCR was performed using the Brilliant III ultra-Fast SYBR green qPCR master mix (Agilent Technologies, catalog #600882) according to the manufacturer’s instructions. Data were normalized to *Gapdh*. The primers used are as follows: *Mbp* forward, GGC ATC ACA GAA GAG ACC CTC; *Mbp* reverse, GCA CCC CTG TCA CCG CTA AAG; *Gapdh* forward, GGG TGT GAA CCA CGA GAA ATA; *Gapdh* reverse, CTG TGG TCA TGA GCC CTT C. All primers were ordered via Integrated DNA Technologies (IDT).

### Slide scanner imaging

Whole slide scans were acquired on a 3DHistech Pannoramic 250 Flash III Slide Scanner using a Zeiss 40 × 0.95 NA objective. The instrument was operated in extended focus mode (seven focal planes spanning a 5-μm axial distance) to capture the entire cell volume across each tissue section. Maximum intensity projections of all the *z*-planes are shown in the specified figures. One image was captured per slide. The microscope is housed in the Imaging Facility at The Hospital for Sick Children in Toronto (ON, Canada). Digital images obtained from the Slide Scanner were imported to the HALO Image Analysis Platform (Indica Labs, v.3.5.3577.173) for subsequent analysis.

### Epifluorescence imaging

Fluorescence images were captured with a 40× water-immersion or 63× oil-immersion objective on a Zeiss Axio Imager.M2 upright microscope. The microscope is housed in the laboratory at The Hospital for Sick Children in Toronto (ON, Canada). Images were evenly captured across Layers II–VI (L2–L6) of the motor and somatosensory cortex on each tissue sample, for a total of 18 images per mouse. All imaging was captured using the Zen Blue software (Carl Zeiss Meditec, v3.3.89.0000). Digital images obtained from the Zeiss Axio Imager.M2 were then imported to the Volocity 3D Image Analysis Software (PerkinElmer, v6.3.1) for subsequent analysis.

### Confocal imaging

High magnification brightfield images for OLIG2/SA-β-gal and PDGFRA/SA-β-gal analysis were acquired using a 40×/1.3 objective on Nikon Eclipse Ti2-E equipped with a Nikon DS10 color camera, running NIS Elements acquisition software. The microscope is housed in the Imaging Facility at The Hospital for Sick Children in Toronto (ON, Canada).

### Quantification of SA-β-gal activity

Two regions of interest (ROIs) were drawn across L2–L6 of the motor and somatosensory cortex on each tissue sample, for a total of six ROIs of equivalent area per mouse. The Multiplex IHC (v3.1.4) algorithm on HALO was used for all cell type specific SA-β-gal activity quantification and was optimized to detect cell type specific positivity based on the respective DAB staining. An ROI of ∼3-mm^2^ spanning the motor and somatosensory cortical regions was drawn for quantification of cell counts in the cortex across all biological replicates. A cytoplasmic radius was then measured and the percentage of cells that exhibited SA-β-gal positivity within the nuclear or associated cytoplasmic compartments was calculated.

### Quantification of PDGFRA/CC1/OLIG2 and PDGFRA/KI67 co-localization

The HighPlex FL (v4.1.3) algorithm on HALO was used for quantification of PDGFRA/OLIG2/CC1 and KI67/PDGFRA colocalization positivity. The module was optimized to detect OLIG2 or PDGFRA-positive nuclear positivity for quantification of PDGFRA/CC1/OLIG2 and KI67/PDGFRA cell counts, respectively. A cytoplasmic radius was measured and the percentage of cells that exhibited concomitant nuclear or cytoplasmic compartment positivity for other markers was calculated accordingly. Thresholds for nuclear and cytoplasmic positivity were calibrated for each marker set and slide. An ROI of ∼3-mm^2^ spanning L2–L6 of the motor and somatosensory cortical regions was drawn for quantification of cell counts in the cortex; an ROI of ∼1 mm^2^ was drawn medially along the corpus callosum (CC) for quantification of cell counts in the CC for all biological replicates.

### Quantification of nuclear LMNB1 fluorescence intensity

A total of 100 PDGFRA-positive cells were imaged from Ts65Dn, Ts65Dn+fisetin, or CTL mice (*n *=* *3 per condition) across the motor and somatosensory cortex. Each image contained at least one PDGFRA-positive cell. PDGFRA-positive cells were manually selected and individually assessed for nuclear LMNB1 colocalization within the imaged plane. The threshold for positive LMNB1 staining was set to ∼1 SD from the mean across all sampled images. Normalized LMNB1 intensity was then calculated for each PDGFRA-positive cell by dividing the obtained LMNB1 intensity value by the total surface area of the associated cell.

### Quantification of MBP fluorescence intensity

Myelin intensity thresholds were established based on parameters that allowed the maximum capture of MBP stain across all images for all sampled conditions. Normalized MBP intensity was then calculated for each image by dividing the obtained MBP intensity value by the total surface area of all myelin sheaths within the imaged frame. All images were captured across L2–L6 of the motor and somatosensory cortex.

### Immunostaining statistical analyses

Statistical significance was evaluated using a two-tailed Student’s *t* test for all experiments evaluating comparisons between three-month Ts65Dn and CTL mice, or using a one-way ANOVA followed by a *post hoc* Tukey’s test for all experiments evaluating comparisons between Ts65Dn, Ts65Dn+fisetin, and CTL genotypes. The statistical analyses were performed on R (The R Project for Statistical Computing, RRID:SCR_001905). Error bars displayed on graphs denote the SD from the mean.

### Tissue preparation for single-nucleus sequencing

For single-nucleus sequencing, primary somatosensory cortical tissue from six-month Ts65Dn and CTL (*n *=* *3 per condition) mice were microdissected and snap frozen in liquid nitrogen. Frozen tissue was thawed in 1 ml buffer HB (0.25 m sucrose, 25 mm KCl, 5 mm MgCl_2_, 20 mm Tricine-KOH pH 7.8, 0.15 mm spermine tetrahydrochloride, 0.5 mm spermidine trihydrochloride, 1 mm DTT). The tissue was transferred to a 7-ml dounce; 340 μl 5% IGEPAL CA-630 (Sigma-Aldrich) and 4 ml HB were added to the tissue and the tissue was homogenized with a tight pestle 10–15 times. The sample was transferred to a 15-ml tube and total solution brought to 10 ml with 50% iodixanol; 1 ml 30% iodixanol was layered on top of 1 ml 40% iodixanol in a separate Corex tube (ThermoFisher). The 9 ml sample was layered on top of the iodixanol cushion. The sample was spun at 10,000 × *g* for 18 min; 1 ml of sample at the 30–40% iodixanol interface was collected. After counting nuclei with a hemocytometer, the sample was diluted to 100,000 nuclei/ml with 30% iodixanol (with RNasin) and subjected to single nuclear droplet encapsulation with the 10× Chromium platform (10× Genomics). Libraries were sequenced using the Illumina NovaSeq 6000 S4 platform at The Center for Applied Genomics (TCAG) at The Hospital for Sick Children in Toronto (ON, Canada).

### Single-nucleus RNA-sequencing (snRNA-seq) bioinformatics workflow

Raw reads were converted to FASTQs, mapped to the mm10 mouse reference genome (Ensembl 93), and demultiplexed to generate a per-cell count matrix using the CellRanger pipeline (10× Genomics). Cells from all snRNA-seq experiments were combined into a single Seurat object dataset and filtered through the Seurat pipeline (v4.3.0; Hao et al., 2021) using default parameters. Low-quality cells with unique feature counts >2500 or <200 and >5% mitochondrial counts were excluded. The data was then log-normalized and scaled by the default scale factor of 10,000. The top variable features and principal components were then calculated using default values, returning 2000 features for the dataset. A second round of scaling shifted the expression of each gene so that mean expression across cells was 0 and variance across cells was 1. Linear dimensional reduction was performed and the dimensionality of the dataset was determined. Clustering was performed by constructing a KNN graph and applying the Louvain algorithm. Dimensional reduction was performed with default values for uniform manifold approximation and projection (UMAP) and individual clusters were annotated based on expression of lineage-specific markers.

### Differential gene expression (DGE) analysis

Differential gene expression (DGE) between the two conditions (Ts65Dn vs CTL) for each cell type was assessed with the Seurat FindMarkers function using default MAST parameters and a log-fold change (logFC) threshold of 0.1. Benjamini–Hochberg corrected *p*-values were used for significance. Genes with false discovery rate (FDR) < 0.05 met statistical criteria for significant differential expression. Major cell type annotations were assigned to clusters by manual inspection of canonical marker gene signals and confirmed through scPred’s reference-based mapping approach (v1.9.2; Alquicira-Hernandez et al., 2019) using the Allen Brain Atlas whole cortex and hippocampus 10× dataset (Yao et al., 2021).

### Ligand-receptor (LR) analysis

Cell-cell communication between all cortical cell types was identified based on ligand-receptor (LR) analysis conducted using CellChat (v1.6.0; S. Jin et al., 2021). Default parameters between the two conditions (Ts65Dn vs CTL) were employed. CellChat infers biologically significant LR pairs by assigning each interaction with a probability value and peforming a permutation test, in which *p* < 0.05 is considered significant.

### Gene set enrichment analysis (GSEA)

GSEA was conducted using clusterProfiler (v3.8; Yu et al., 2012) and the Reactome gene set (Gillespie et al., 2022) for each cell type on a ranked list of all the genes for which log_2_FC between conditions (Ts65Dn vs CTL) values were available. Gene sets with FDR < 0.05 were considered significantly enriched, and normalized enrichment scores (NES) were used to assess the directionality of enrichment. The previously published SenMayo gene set (Saul et al., 2022) was used to perform transcriptomic GSEA for the senescence-associated gene signature and clusterProfiler was used to compute an NES for each cell type. Statistically significant enrichment for the gene set was determined by cell types showing FDR < 0.05. All FDR values were calculated using the Benjamini–Hochberg method.

### Single-nucleus ATAC-sequencing (snATAC-seq) bioinformatics workflow

Cells from all snATAC-seq experiments were combined into a single Seurat object dataset and filtered through the Signac pipeline (v1.10.0; Stuart et al., 2021) using default parameters. Low-quality cells were removed using filters for peak region fragments <100,000, percent reads in peaks >40, blacklist site ratio <0.025, nucleosome signal <4, and transcriptional start site (TSS) enrichment score >2. The data was then normalized via singular value decomposition (SVD) of the term frequency-inverse document frequency (TF-IDF) matrix and all identified variable features were used for linear dimensional reduction. Remaining preprocessing and dimensionality reduction was performed according to Signac recommendations using default parameters. A gene activity matrix was constructed by counting snATAC-seq peaks within the gene body and 2 kb upstream of the transcriptional start site (TSS) using protein-coding genes annotated in the Ensembl database. UMAP dimensionality reduction was performed with default values.

### Differential accessibility analysis

Differentially accessible regions (DARs) between the two conditions (Ts65Dn vs CTL) for each cell type were calculated with the Signac FindMarkers function using default MAST parameters and a log-fold change (logFC) threshold of 0.1 and n_count_peaks as a latent variable. Benjamini–Hochberg corrected *p*-values were used for significance. Peaks with FDR < 0.05 met statistical criteria for significant differential accessibility. Major cell type annotations were assigned to clusters through manual inspection of canonical marker gene signals and confirmed through label transfer using Seurat’s multimodal snRNA-seq to snATAC-seq integration pipeline. Integration showed 100% cluster label concordance between the two modalities.

### Gene ontology (GO) analysis

Gene ontology (GO) analysis was performed for all enriched genes (FDR < 0.05) in each cell type using g:Profiler (v0.7.0; Raudvere et al., 2019) to identify overrepresentation for biological process (BP) and molecular function (MF) categories. Statistically significant enrichment for GO pathways was determined by cell types showing FDR < 0.05. All FDR values were calculated using the Benjamini–Hochberg method.

### Transcription factor (TF) motif analysis

Transcription factor (TF) motif activity was estimated using the RunChromVAR wrapper in Signac. The positional weight matrix (PWM) was obtained from the JASPAR2020 database (Fornes et al., 2020). Differential motif activity was assessed using log_2_fold change (log_2_FC) threshold of 0.1 with a 0 pseudocount. Motifs with FDR < 0.05 met statistical criteria for significant binding activity.

### Fear conditioning

Behavioral testing was performed at The Centre for Phenogenomics (TCP) in Toronto (ON, Canada). Animals were left in their home cage inside the testing room, anteroom or on the day-rack at least 30 min before testing. Mice were then transferred into individual testing chambers with grid floors. After 120 s without any stimulus (which gives the mouse time to get used to the surrounding) a 30 s, 3600 kHz 95-dB audible tone (CS, conditioned stimulus) followed and was co-terminated with a 2-s foot shock of 0.75 mA (US, unconditioned stimulus). Following the shock, mice spent another 30 s in the chamber before removal. Concomitantly, the animals associated the background context cues with the CS (conditioning). Mice were then returned to their holding room where they spent 24 h before being returned to the testing room for additional screening. They were placed back into the same testing chambers for 300 s without changes to the context/interior and no stimuli applied. After conditioning, the CS or the spatial context elicited, in the absence of the US, a central state of fear that was expressed as reduced locomotor activity or total lack of movement (freezing). The time spent immobile was used as a measure of learning/memory performance. This phase was followed by a 2 h break, which mice spent in their home cage before being returned to the testing chamber with a changed context. The grid floor was covered with a plastic sheet, the ceiling was changed from a square to triangular shape, the olfactory cues were changed, and the noise levels were reduced. Mice spent 180 s in this novel environment before a 180 s 3600 kHz 95-dB audible tone (no foot shock) was played again. Following this phase animals, were placed back into their home cage and the test was completed. Clidox 1:5:1 and 70% EtOH solutions were used to disinfect surfaces between testing mice from different cages.

### Replicates

All immunostaining analyses were performed on at least three different brains each. All single-nucleus sequencing analyses were performed on three different brains each. All behavioral analyses were performed on at least eight different mice each. Additional details of the statistical analyses can be found in the detailed descriptions of the immunostaining and computational methods above and, where relevant, in the results and the figures legends. For all analyses, *n* refers to number of animals analyzed.

### Data availability

Sequencing data have been deposited in GEO under accession code GSE225554. All data generated or analyzed during this study are included in the manuscript and supporting files. The custom code used for this manuscript is available on GitHub at https://github.com/KalishLab/Ts65Dn_OPC_Senescence.

## Results

### Characterization of cell types in the adult Ts65Dn cortex using multiomic single-nucleus sequencing

**Figure 1.**
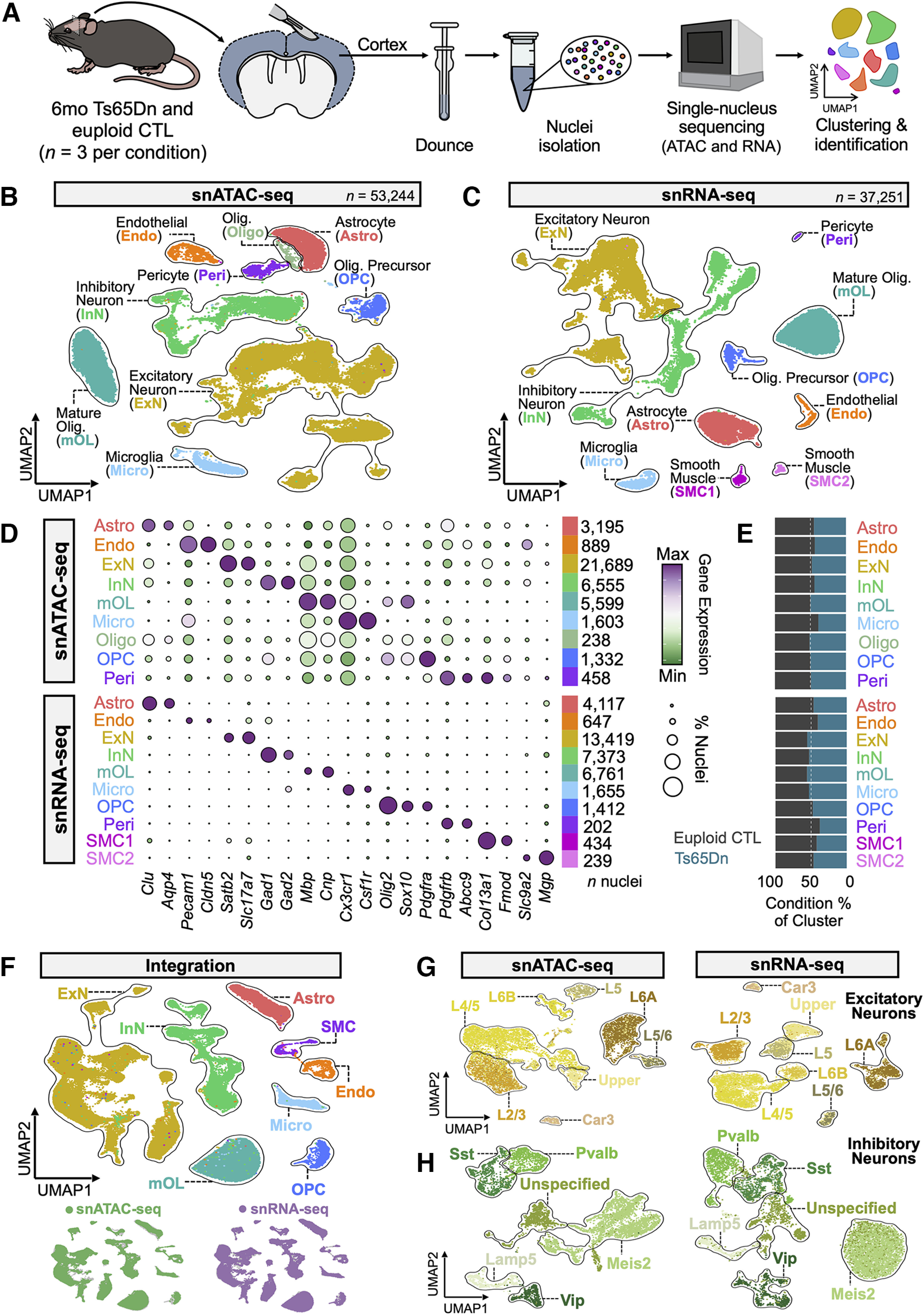
Multimodal single-nucleus sequencing of the Ts65Dn mouse cortex identifies all major neuronal and non-neuronal cell types. ***A***, Schematic representation of biological samples, cortical dissection, tissue processing and sequencing workflow for snATAC-seq and snRNA-seq from six months Ts65Dn and euploid control (CTL) mice (*n* = 3 male mice per condition). ***B***, UMAP visualization of the snATAC-seq dataset, where each dot represents a single nucleus, for a total of 53,244 nuclei. UMAP plots are generated from combined replicates across Ts65Dn and CTL conditions. Each cluster is colored by cell type: Astro, astrocytes; Endo, endothelial cells; ExN, excitatory neurons; InN, inhibitory neurons; mOL, mature oligodendrocytes; Micro, microglia; Oligo, oligodendrocytes; OPC, oligodendrocyte precursor cells; Peri, pericytes; SMC1/2, smooth muscle cells. ***C***, As in B, but a visualization of the snRNA-seq dataset, containing a total of 37,251 nuclei. ***D***, Dotplot of snATAC-seq and snRNA-seq datasets showing gene expression or gene accessibility patterns, respectively, with several key canonical marker genes used for cluster identification. The diameter of the dot corresponds to the proportion of nuclei expressing or exhibiting accessibility of the indicated gene, and the color of the dot corresponds to the average expression or accessibility of the gene relative to all cell types. The number of nuclei assigned to each cell type are indicated. ***E***, Barplot depicting the fraction of nuclei per cell type by condition. ***F***, UMAP visualization of multi-omic integration of snATAC-seq and snRNA-seq datasets colored by cell type assignment. Beneath are UMAPs of the integration colored by originating dataset. ***G***, UMAP visualization of excitatory neuron (ExN) subsets as extracted from snATAC-seq and snRNA-seq datasets. Each cluster is colored by cell type: Car3, Car3-expressing excitatory neurons; L2/3, cortical Layers II -III; L4/5, cortical Layers IV -V; L5, cortical Layer V; L5/6, cortical Layers V -VI; L6A/B, cortical Layer VI excitatory neuron subsets; Upper, mixed upper layer (II -IV) excitatory neurons. ***H***, As in ***G***, but a visualization of inhibitory neuron (InN) subsets. Each cluster is colored by cell type: Lamp5, Lamp5-expressing interneurons; Meis2, Meis2-expressing interneurons; Pvalb, parvalbumin-expressing interneurons; Sst, somatostatin-expressing interneurons; Vip, vasoactive intestinal peptide-expressing interneurons; Unspecified, interneurons of unspecified classification. See Extended Data Figure 1-1 for quality control (QC) metrics used in the initial processing of snATAC-seq and snRNA-seq data.

To identify cellular programs underlying age-related cognitive decline in trisomy, we performed droplet-based single-nucleus ATAC (snATAC-seq) and RNA-sequencing (snRNA-seq) on the six-month Ts65Dn and euploid control (CTL) littermate mouse cortex using the 10× Genomics platform (Fig. 1*A*; Materials and Methods; G.X.Y. Zheng et al., 2017; Satpathy et al., 2019). Nuclei from each biological sample were split into two fractions to capture and analyze both the mRNA and accessible chromatin: one part was used for snATAC-seq and the other was used for snRNA-seq. Ts65Dn mice and littermate controls were selected from three separate litters. This study was restricted to the cortex, the outermost portion of the brain, because of its involvement in cognition and higher-order processing (Shipp, 2007), and the associated structural and functional deficits known to occur in DS (N.R. Lee et al., 2016; Utagawa et al., 2022). We analyzed the transcriptomes and epigenomes from each dataset using the well-established Seurat (Hao et al., 2021) and Signac (Stuart et al., 2021) pipelines, respectively (Materials and Methods). Data was quality control (QC) filtered to remove clusters likely to be of low quality resulting from debris, doublets, or dead cells, and was subsequently normalized using default parameters. After QC processing, we profiled a total of 53,244 nuclei for snATAC-seq and 37,251 nuclei for snRNA-seq (Extended Data Fig. 1-1).

10.1523/ENEURO.0147-23.2023.f1-1 Extended Data Figure 1-1 Quality control (QC) metrics for multiomic single-nucleus data and integration of snATAC-seq and snRNA-seq data for neuronal subsets, related to Figure 1. ***A***, Quality control (QC) metrics for snATAC-seq data, including the percentage of reads in peaks, the number of peak region fragments, transcription start site (TSS) enrichment, blacklist ratio, and nucleosome signal per replicate from six-month Ts65Dn and euploid control (CTL) mice (*n *=* *3 male mice per condition). ***B***, As in ***A***, but depicting QC metrics for snRNA-seq data, including the number of features, total transcript counts, and percent mitochondrial content per replicate. ***C***, UMAP visualization of multiomic integration of snATAC-seq and snRNA-seq datasets colored by originating dataset for the excitatory neuron (ExN) subset. ***D***, As in ***C***, but depicting multiomic integration for the inhibitory neuron (InN) subset. Download Figure 1-1, TIF file.

Genes with high variance were used to compute principal components for projecting and clustering cell populations with similar molecular signatures. Unsupervised clustering through Leiden clustering and plotting via uniform manifold approximation and projection (UMAP; Materials and Methods) dimensionality reduction revealed 10 major cell classes across the snATAC-seq and snRNA-seq datasets: astrocytes (Astro), excitatory neurons (ExN), inhibitory neurons (InN), mature oligodendrocytes (mOL), microglia (Micro), oligodendrocyte precursor cells (OPC), oligodendrocyte lineage cells (Oligo), endothelial cells (Endo), pericytes (Peri), and smooth muscle cells (SMC; Fig. 1*B*). Cell types were assigned based on a combination of canonical marker gene expression (Fig. 1*C*) and confirmed through a machine learning reference-based mapping approach (scPred; Materials and Methods; Alquicira-Hernandez et al., 2019) using the Allen Brain Atlas whole cortex and hippocampus 10× Genomics dataset (Materials and Methods; Yao et al., 2021). Integration and label transfer (Materials and Methods) between snRNA-seq and snATAC-seq further confirmed cell population assignments and demonstrated strong concordance between the two modalities, with cell types identified in either platform grouping together in the integrated UMAP space (Fig. 1*D*). All major cell types were present in both conditions, with high transcriptomic and epigenetic overlap between genotypes.

The ExN and InN clusters were proportionally the largest by number out of all identified cell type clusters, consistent with previous reports that neurons are the most numerous cell type in the cortex (Keller et al., 2018). To further subcategorize heterogeneous neuronal populations, we performed a second level of hierarchical clustering on ExNs and InNs to identify distinct subpopulations of each neuron class (Fig. 1*E*). Again, using the scPred algorithm on the snRNA-seq dataset, we identified clusters spanning five cortical layers (L2–L6), a distinct Car3+ population of ExNs, as well as five subpopulations of InNs, all with distinct transcriptional signatures. Integration of snRNA-seq and snATAC-seq neuronal subclusters revealed corresponding ExN cortical layer and InN subpopulations within the snATAC-seq dataset (Extended Data Fig. 1-1). Overall, this analysis transcriptionally and epigenetically identified all major neuronal and non-neuronal cell types and subtypes across the murine cortex.

### Identification of cell type-specific trisomy-associated transcriptomic changes in the Ts65Dn cortex

**Figure 2.**
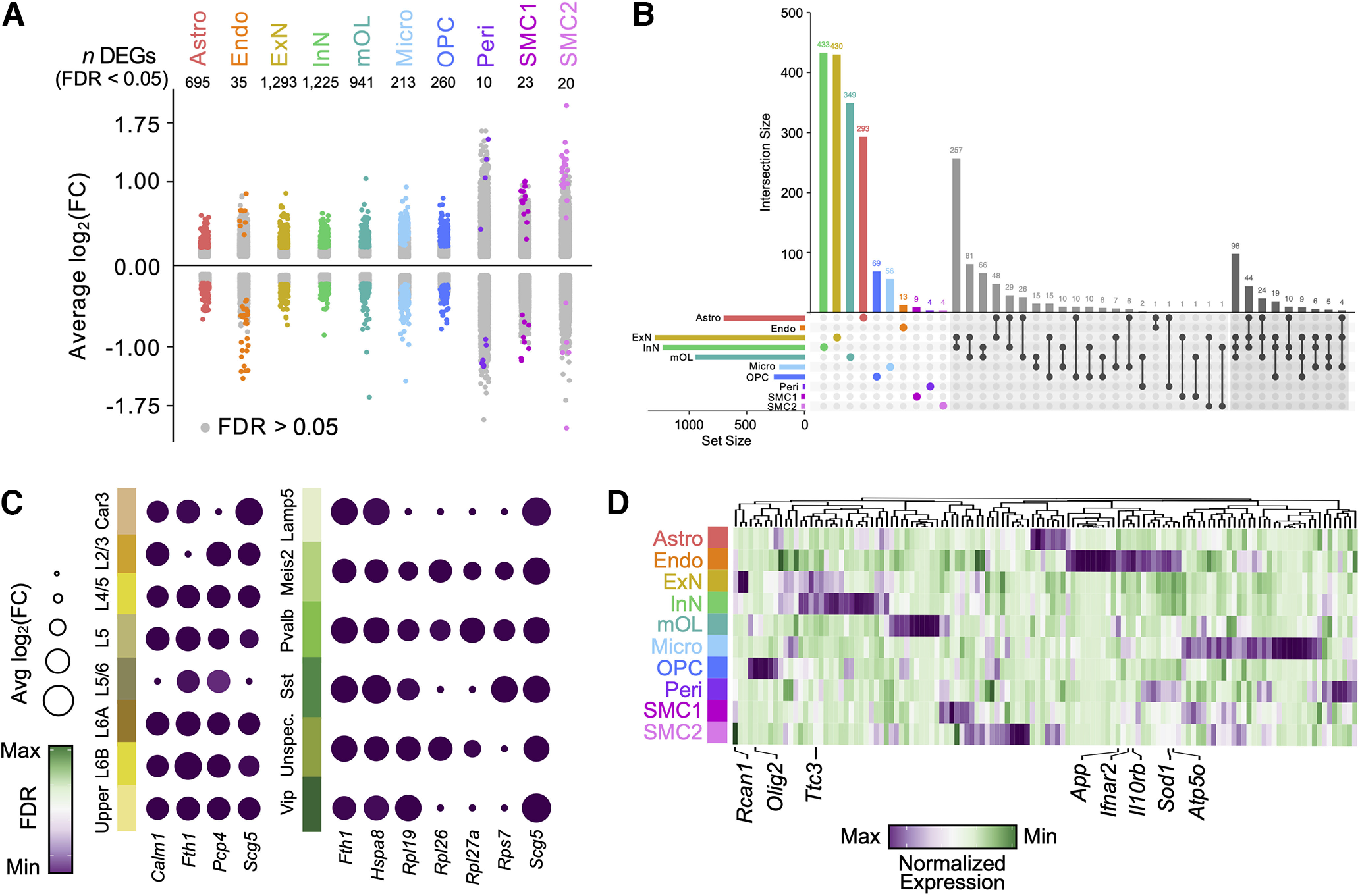
Transcriptionally distinct cellular subpopulations in Ts65Dn cortex. ***A***, Strip plot displaying differentially expressed genes (DEGs) between Ts65Dn and CTL offspring at six months. Colored dots represent significant genes (FDR < 0.05) per cell type. The number of significant DEGs per cell type is indicated. The *x*-axis displays all major cortical cell types profiled through snRNA-seq. Data obtained from *n *= 3 biological replicates per condition. ***B***, UpSet plot displaying the number of unique and shared DEGs across cell types, with unique genes colored based on cell type, and genes shared between two or three cell types indicated by black dots connected by lines according to shared origins. The histogram indicates the number of DEGs for each cell type, and the barplots show the number of significant DEGs (FDR < 0.05) per cell type. ***C***, Dotplot of select differentially expressed genes (DEGs) within excitatory neuron (ExN) and inhibitory neuron (InN) subsets. Each row represents a neuronal subset population. Each column represents a gene. The color code represents the FDR, and the size of the dots represents the average log_2_fold change [avg log_2_(FC)] of gene expression between Ts65Dn and CTL mice. ***D***, Hierarchically clustered heatmap of normalized gene expression for genes contained within the Ts65Dn chromosomal product. The plot displays gene activity across all cell types in snRNA-seq. Several genes of interest are indicated. Each column represents a cell type. The color code represents the row-normalized expression for each gene.

To identify transcriptional signatures of trisomy-associated cognitive decline, we performed cell type-specific differential gene expression (DGE) between Ts65Dn and euploid CTL mice (Materials and Methods). We used differentially expressed genes (DEGs) that met a false discovery rate (FDR) value < 0.05 to define statistically significant changes in transcript expression between Ts65Dn and CTL offspring. We identified a total of 4715 cell type-specific DEGs across all cell populations (Fig. 2*A*; Table 1), with 64.3% of DEGs upregulated (log_2_FC > 0) and 35.7% downregulated (log_2_FC < 0) in Ts65Dn. We found that 38% of the DEGs significantly altered by Ts65Dn trisomy were cell type specific, with the remaining 62% intersecting between two or more cell types (Fig. 2*B*).

**Table 1:** Top five genes enriched in each cortical cell type in Ts65Dn mice compared with CTL

Gene	FDR	log_2_(FC)	Cell type	Gene	FDR	log_2_(FC)	Cell type
*Scg5*	1.66E-19	0.55	Astro	*Scg5*	3.20E-09	0.86	Micro
*mt-Co3*	3.08E-19	0.51	Astro	*Pcp4*	1.87E-17	0.74	Micro
*Pcp4*	3.56E-23	0.48	Astro	*Fmn1*	5.78E-04	0.70	Micro
*Fth1*	1.98E-28	0.47	Astro	*Hbb-bs*	1.36E-04	0.64	Micro
*mt-Co1*	9.46E-10	0.46	Astro	*Rpl26*	7.83E-06	0.60	Micro
*Nxpe2*	1.55E-08	1.13	Endo	*Fth1*	8.83E-13	0.73	OPC
*H2-K1*	1.32E-04	0.78	Endo	*mt-Co1*	3.23E-08	0.66	OPC
*Ly6a*	4.38E-02	0.60	Endo	*Ano3*	4.03E-07	0.65	OPC
*Ccni*	1.03E-02	0.60	Endo	*mt-Nd4*	1.21E-05	0.57	OPC
*Nxpe4*	4.07E-02	0.60	Endo	*mt-Cytb*	1.01E-05	0.55	OPC
*mt-Co3*	1.40E-234	0.79	ExN	*Adora2a*	2.35E-01	1.47	Peri
*Fth1*	3.68E-196	0.64	ExN	*Tnfrsf21*	2.58E-01	1.47	Peri
*mt-Co1*	2.18E-136	0.64	ExN	*Nefl*	4.06E-02	1.38	Peri
*Pcp4*	1.29E-125	0.57	ExN	*Arpc2*	9.73E-02	1.36	Peri
*Calm1*	2.77E-212	0.55	ExN	*Anp32b*	2.58E-01	1.31	Peri
*Scg5*	5.85E-70	0.57	InN	*Sdc2*	1.03E-02	0.92	SMC1
*Gpr88*	1.53E-24	0.51	InN	*Tuba4a*	1.03E-02	0.91	SMC1
*Hsp90aa1*	1.45E-63	0.49	InN	*Arpp19*	1.03E-02	0.87	SMC1
*Fth1*	8.98E-71	0.49	InN	*Uqcrh*	4.07E-04	0.87	SMC1
*Ckb*	5.74E-65	0.48	InN	*Tpt1*	5.12E-03	0.82	SMC1
*Spock1*	1.01E-61	0.94	mOL	*mt-Co2*	1.97E-06	1.74	SMC2
*Fmn1*	1.41E-22	0.66	mOL	*Pcp4*	5.18E-09	1.34	SMC2
*Hbb-bs*	6.47E-18	0.60	mOL	*mt-Co3*	2.13E-04	1.24	SMC2
*Pcp4*	8.02E-49	0.58	mOL	*Rps20*	3.17E-02	1.20	SMC2
*Camk2n1*	6.42E-19	0.53	mOL	*Scand1*	6.83E-03	1.19	SMC2

Listed are the top five genes [ranked by highest average log_2_(FC)] that are significantly differentially enriched in Ts65Dn mice compared with CTL as detected via snRNA-seq. Shown are fold-change (FC) values of the average expression for each gene and the false discovery rate (FDR) between Ts65Dn and CTL cells. Each cell type is listed in the final column: Astro, astrocytes; Endo, endothelial cells; ExN, excitatory neurons; InN, inhibitory neurons; mOL, mature oligodendrocytes; Micro, microglia; OPC, oligodendrocyte precursor cells; Peri, pericytes; SMC1/2, smooth muscle cells.

ExN and InN populations were the most numerous of all identified cell types, and accordingly had the highest number of total and shared DEGs (Fig. 2*B*). Several genes were found to be overexpressed in a majority of ExN subclusters (at least five out of eight; Fig. 2*C*; Table 2), including *Calm1*, an essential regulator of early neuronal migration (Kobayashi et al., 2015), *Fth1*, a ferroxidase enzyme that supports iron detoxification as part of the neuronal antioxidant defense system (Mukherjee et al., 2020), *Scg5*, a secretory chaperone that co-localizes with aggregated proteins in neurodegenerative diseases (Chaplot et al., 2020), and *Pcp4*, a modulator of calcium signaling that is triplicated in DS (Mouton-Liger et al., 2011). A similar trend was observed in InNs, with several genes showing overexpression across multiple subtype clusters (at least four out of six; Fig. 2*C*), including the aforementioned *Fth1* and *Scg5*, as well as *Hspa8*, a molecular chaperone whose expression increases during injury and activates proinflammatory responses through NF-κB signaling (Mi et al., 2021). Several large (*Rpl*) and small (*Rps*) ribosomal subunit genes, including *Rpl19/26/27a* and *Rps7* were also overexpressed in several InN subtypes, suggesting a disruption in translation-associated ribosomal machinery. These data support existing findings of metabolic (Izzo et al., 2018; Dierssen et al., 2020; Sarver et al., 2023), inflammatory (Wilcock, 2012; Flores-Aguilar et al., 2020), transcriptional (Gardiner, 2006), and translational (Sullivan et al., 2016; Aivazidis et al., 2017) dysregulation in the trisomic brain.

**Table 2:** Top five genes enriched in each excitatory (ExN) and inhibitory (InN) neuronal subtype in Ts65Dn mice compared with CTL

Gene	FDR	log_2_(FC)	Cell type	Gene	FDR	log_2_(FC)	Cell type
Scg5	1.49E-03	0.92	Car3	*Fth1*	1.54E-47	0.64	Upper
*Bex2*	1.52E-03	0.86	Car3	*Pcp4*	1.27E-33	0.58	Upper
*Rtn1*	1.13E-08	0.84	Car3	*Calm1*	6.98E-47	0.55	Upper
*Cck*	7.34E-03	0.79	Car3	*Scg5*	2.40E-31	0.55	Upper
*Tomm20*	3.67E-03	0.73	Car3	*Cmss1*	6.98E-47	0.48	Upper
*Pcp4*	5.10E-26	0.68	L2/3	*Cck*	4.04E-02	0.71	Lamp2
*Calm1*	8.56E-42	0.61	L2/3	*Pcp4*	7.90E-04	0.69	Lamp2
*Fth1*	2.51E-25	0.59	L2/3	*Scg5*	8.38E-03	0.67	Lamp2
*Cd81*	4.06E-18	0.58	L2/3	*Tac1*	1.61E-02	0.67	Lamp2
*Lama2*	2.33E-04	0.56	L2/3	*Vdac2*	1.73E-02	0.63	Lamp2
*Fth1*	1.65E-46	0.61	L4/5	*Scg5*	3.52E-31	0.59	Meis2
*Pcp4*	1.16E-32	0.58	L4/5	*Junb*	2.23E-14	0.57	Meis2
*Camk2n1*	9.50E-29	0.54	L4/5	*Gpr88*	2.90E-15	0.48	Meis2
*Scg5*	7.07E-30	0.52	L4/5	*Ckb*	1.89E-34	0.46	Meis2
*Pld5*	3.36E-18	0.52	L4/5	*Actb*	2.12E-19	0.46	Meis2
*Fth1*	1.47E-13	0.71	L5	*Pcp4*	3.43E-11	0.67	Pvalb
*Camk2n1*	1.09E-08	0.69	L5	*Chchd2*	2.53E-09	0.58	Pvalb
*Itm2c*	1.19E-09	0.65	L5	*Fth1*	1.00E-08	0.57	Pvalb
*Calm1*	5.88E-15	0.63	L5	*Scg5*	2.60E-07	0.56	Pvalb
*Ubb*	1.75E-11	0.62	L5	*Actg1*	9.05E-09	0.56	Pvalb
*Eid1*	3.14E-03	0.79	L5/6	*Hspa8*	5.86E-08	0.67	Sst
*Tuba1a*	2.50E-03	0.78	L5/6	*Itm2c*	4.58E-06	0.63	Sst
*Lars2*	3.83E-02	0.63	L5/6	*Pcp4*	3.38E-09	0.61	Sst
*Eef1g*	2.59E-02	0.62	L5/6	*Fth1*	1.31E-07	0.60	Sst
*Ndufa13*	3.83E-02	0.61	L5/6	*Scg5*	1.16E-06	0.59	Sst
*Fth1*	4.00E-37	0.71	L6A	*Gpr88*	2.66E-08	0.56	Unspec
*Calm1*	5.38E-40	0.62	L6A	*Cck*	2.94E-04	0.56	Unspec
*Atp6v0c*	1.97E-22	0.61	L6A	*Fth1*	5.39E-17	0.54	Unspec
*Tubb5*	3.17E-19	0.57	L6A	*Scg5*	1.15E-10	0.52	Unspec
*Serinc1*	4.53E-17	0.54	L6A	*Ckb*	1.12E-14	0.48	Unspec
*Fth1*	1.32E-14	0.93	L6B	*Scg5*	5.01E-08	0.72	Vip
*Rpl23*	4.66E-05	0.68	L6B	*Vip*	7.19E-06	0.71	Vip
*Pgrmc1*	6.01E-05	0.66	L6B	*H3f3b*	1.29E-07	0.63	Vip
*Rpl13*	4.36E-07	0.62	L6B	*Lars2*	3.34E-04	0.57	Vip
*Rpl26*	9.65E-06	0.61	L6B	*Rpl19*	5.11E-05	0.55	Vip

Listed are the top five genes [ranked by highest average log_2_(FC)] that are significantly differentially enriched in Ts65Dn mice compared with CTL as detected via snRNA-seq. Shown are fold-change (FC) values of the average expression for each gene and the false discovery rate (FDR) between Ts65Dn and CTL cells. Each neuronal subtype is listed in the final column: Car3, Car3-expressing excitatory neurons; L2/3, cortical Layers II–III; L4/5, cortical Layers IV–V; L5, cortical Layer V; L5/6, cortical Layers V–VI; L6A/B, cortical Layer VI excitatory neuron subsets; Upper, mixed upper layer (II-IV) excitatory neurons; Lamp5, Lamp5-expressing interneurons; Meis2, Meis2-expressing interneurons; Pvalb, parvalbumin-expressing interneurons; Sst, somatostatin-expressing interneurons; Vip, vasoactive intestinal peptide-expressing interneurons; Unspecified, interneurons of unspecified classification.

All cell types exhibited upregulation of ∼10–20% of the genes expressed on the Ts65Dn trans-chromosome at the transcript level. However, not all Ts65Dn triplicated genes were equally altered in each cell type (Fig. 2*D*), consistent with previous analyses conducted on human iPSC-derived DS lines (Jose Luis Olmos-Serrano et al., 2016; Ponroy Bally et al., 2020) and single-cell analyses of DS mouse models (Lyle et al., 2004; Palmer et al., 2021; Sierra et al., 2021) that demonstrate limited overexpression of triplicated genes in trisomic cells. Only a few genes such as *Atp5o* or *Sod1* were consistently found at higher levels in trisomic mice across various cell types, while others were overexpressed only in particular cell populations. For instance, *Olig2*, a key transcription factor (TF) that is triplicated in DS and activates the expression of myelin-associated genes in OL-lineage cells (Zhou et al., 2000; Zhou and Anderson, 2002), was selectively upregulated in trisomic OPCs by 15%; its overexpression is known to contribute to impaired OPC proliferation and differentiation in DS (Chakrabarti et al., 2010; Jose Luis Olmos-Serrano et al., 2016; Xu et al., 2019). *Ttc3*, an interactor of nerve growth factor (Ngf) that strongly inhibits neurite extension on overexpression (Berto et al., 2007), was upregulated in Ts65Dn ExNs by 13%. *App*, an integral membrane protein that is triplicated in DS and associated with increased amyloid-β (Aβ) aggregation and plaque deposition (O’Brien and Wong, 2011) showed enrichment of 8.6% and 11% in trisomic ExNs and microglia, respectively, and *Rcan1*, whose overexpression leads to neurofibrillary tangles (Ermak and Davies, 2013), was enriched in Ts65Dn endothelial cells by 16%. Lastly, pericytes and astrocytes exhibited an increase in *Ifnar2* expression by 37% and 8.6%, respectively, while microglia showed an increased expression of *Ifnar2* by 10% and *Il10rb* by 13%; the upregulated expression of these IFN receptors may contribute to the hyper-inflammatory milieu of the trisomic brain. The cell type-specific DEGs resulting from trisomy are consistent with many of the molecular disruptions in DS, including impaired myelinogenesis (Ábrahám et al., 2012; Flores-Aguilar et al., 2020), neuroinflammation (Wilcock, 2012; Flores-Aguilar et al., 2020), and neurogenesis (Stagni et al., 2018).

### Gene set enrichment analysis (GSEA) reveals dysregulation of several biological pathways in the mature Ts65Dn cortex

**Figure 3.**
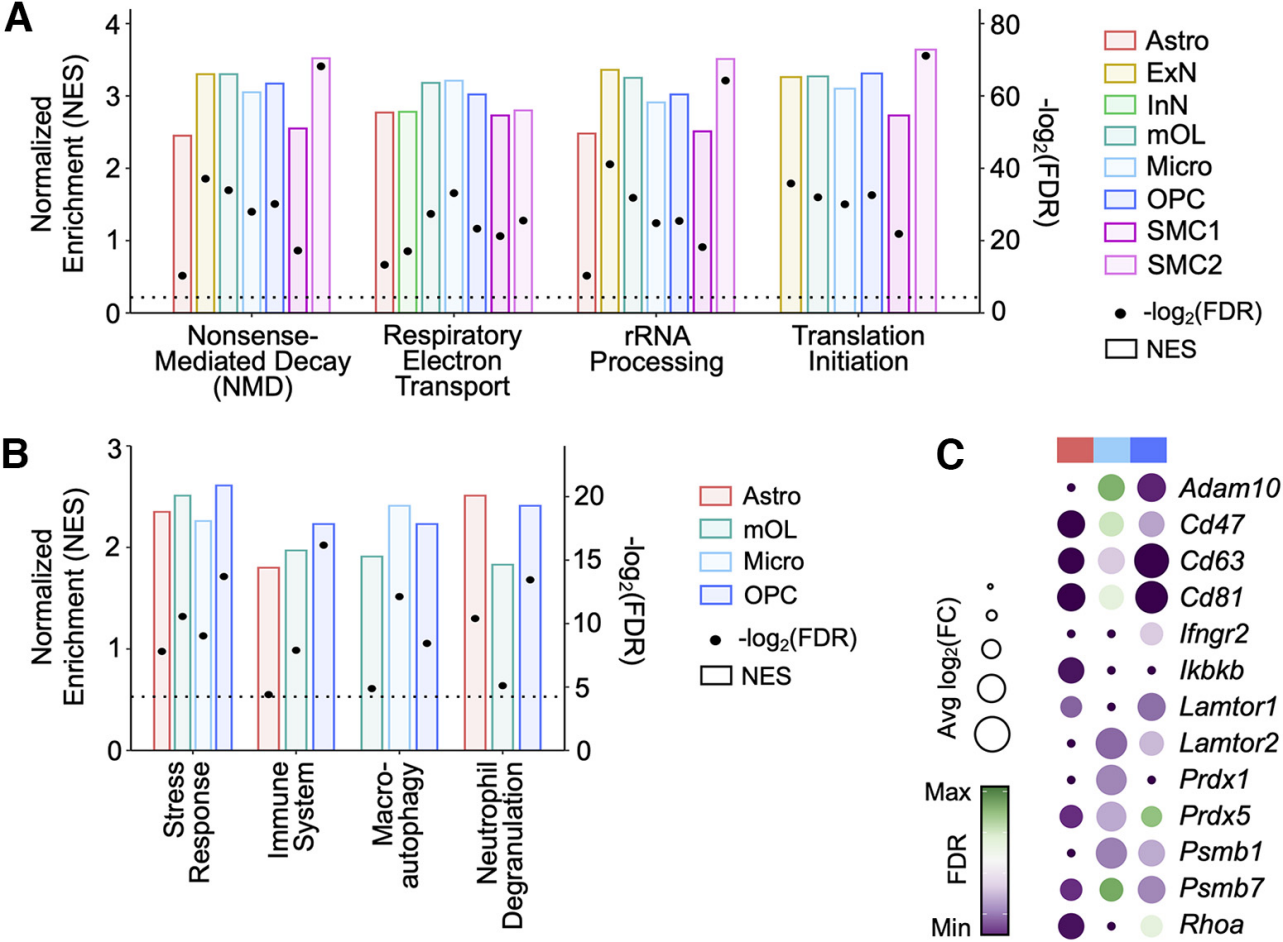
Gene set enrichment analysis (GSEA) reveals disruption of several biological pathways in trisomic cells from the Ts65Dn cortex. ***A***, Gene set enrichment analysis (GSEA) of several Reactome biological pathways enriched across a majority of cortical cell types. The bars correspond to the left *y*-axis, displaying normalized enrichment score for each cell type by pathway and are colored by cell type identity. The dots correspond to the right *y*-axis, displaying -log_2_(FDR) for each cell type by pathway; the dashed horizontal line intercepts the right *y*-axis at 4.3, corresponding to an FDR = 0.05. All dots above this horizontal line are statistically significant. ***B***, As in ***A***, but depicting GSEA of several Reactome biological pathways enriched across astrocytes (Astro), mature oligodendrocytes (mOL), microglia (Micro), and oligodendrocyte precursor cells (OPC). ***C***, Dotplot of select differentially expressed genes (DEGs) within astrocytes (red), microglia (light blue), and OPCs (dark blue). Each column represents a cell population. Each row represents a gene. The color code represents the FDR, and the size of the dots represents the average log_2_fold change [avg log_2_(FC)] of gene expression between Ts65Dn and CTL.

To identify functional pathways perturbed in Ts65Dn mice, we performed gene set enrichment analysis (GSEA) using the Reactome repertoire of biological pathways (Gillespie et al., 2022) on ranked genes across all cell types (Materials and Methods). Of the top 10 enriched GSEA categories, four emerged as common processes altered in almost all cell types: ribosomal RNA (rRNA) processing, respiratory electron transport, nonsense-mediated decay (NMD), and translation initiation (Fig. 3*A*; Table 3). DS is characterized by an impairment in mitochondrial and ribosomal biogenesis, as well as integrated stress response (ISR)-mediated disruption in proteostasis (Aivazidis et al., 2017; Izzo et al., 2018; P.J. Zhu et al., 2019). Increased ribosomal and rRNA processing have been described in DS (Ravaioli et al., 2022) and premature aging (Buchwalter and Hetzer, 2017), and ribosome biogenesis has been shown to be regulated, and thus disrupted, in an ISR-dependent manner (Szaflarski et al., 2022). Consistent with this finding, we observed overexpression and dysregulation of over 30 large (*Rpl*) and small (*Rps*) ribosomal subunit genes and 15 mitochondrial (*mt* and *cox*) transcripts in the Ts65Dn brain. Genes involved in translation initiation, such as *eIF4e, eIF3e*, and *eIF3a* (Gingras et al., 1999; Aitken et al., 2016), were also found to have increased expression in all cortical Ts65Dn cells. Disrupted mitochondrial biogenesis in DS has previously been shown to impair oxidative phosphorylation (Bayona-Bafaluy et al., 2021), reduce mitochondrial energy production, and lower mitochondrial function (Helguera et al., 2013; Bayona-Bafaluy et al., 2021), together resulting in a hypoxia-like neuropathology (Pecze et al., 2020), further suggestive of a widespread metabolic dysregulation in the DS brain.

**Table 3:** Top three Ts65Dn enriched pathways in each cortical cell type identified via gene set enrichment analysis (GSEA) in Ts65Dn mice compared with CTL

GSEA pathway	FDR	NES	Cell type
The citric acid (TCA) cycle and respiratory electron transport	3.51E-05	2.81	Astro
Respiratory electron transport	9.86E-05	2.77	Astro
Nonsense mediated decay (NMD)	1.54E-04	2.65	Astro
Interferon signaling	1.68E-02	2.25	Endo
Interferon-α/β signaling	2.89E-02	2.23	Endo
Negative regulation of the PI3K/AKT network	4.20E-02	2.10	Endo
SRP-dependent cotranslational protein targeting to membrane	4.60E-14	3.48	ExN
Nonsense mediated decay (NMD)	3.06E-13	3.43	ExN
rRNA processing in the nucleolus and cytosol	4.20E-13	3.36	ExN
Metabolism	8.47E-12	3.13	InN
The citric acid (TCA) cycle and respiratory electron transport	2.20E-08	3.04	InN
Innate immune system	3.87E-08	2.94	InN
The citric acid (TCA) cycle and respiratory electron transport	1.57E-11	3.43	mOL
SRP-dependent cotranslational protein targeting to membrane	1.39E-11	3.42	mOL
Nonsense mediated decay (NMD)	1.68E-11	3.39	mOL
The citric acid (TCA) cycle and respiratory electron transport	7.15E-12	3.24	Micro
Respiratory electron transport	1.06E-10	3.21	Micro
Eukaryotic translation initiation	9.00E-10	3.10	Micro
SRP-dependent cotranslational protein targeting to membrane	1.56E-10	3.34	OPC
Eukaryotic translation initiation	1.56E-10	3.31	OPC
Nonsense mediated decay (NMD)	3.06E-10	3.28	OPC
Cellular response to heat stress	5.14E-03	1.84	Peri
Translation	2.05E-03	1.72	Peri
Cellular response to stimuli	1.53E-03	1.71	Peri
Formation of a pool of free 40S subunits	1.63E-07	2.86	SMC1
Nonsense mediated decay (NMD)	1.63E-07	2.84	SMC1
Eukaryotic translation initiation	2.62E-07	2.73	SMC1
SRP-dependent cotranslational protein targeting to membrane	7.11E-24	3.78	SMC2
Nonsense mediated decay (NMD)	1.86E-23	3.74	SMC2
Formation of a pool of free 40S subunits	9.16E-24	3.70	SMC2

Listed are the top three pathways identified via GSEA analysis (ranked by lowest FDR value) that are significantly enriched in Ts65Dn mice compared with CTL. All normalized enrichment scores (NES) are listed in the third column. Each cell type is listed in the final column and is abbreviated as described in Table 1.

DEGs in trisomic astrocytes, microglia, and OPCs also exhibited enrichment of GSEA categories including immune activation, neutrophil degranulation, macroautophagy, and stress response (Fig. 3*B*). In Ts65Dn astrocytes, we observed upregulation of *Ikbkb*, an activator of the NF-κB pathway (Israel, 2010), *RhoA*, a regulator of reactive astrocyte dynamics (Renault-Mihara and Okano, 2017), and inflammatory mediators *Cd47*, *Cd63*, and *Cd81* (Hazrati et al., 2022; Fig. 3*C*). Ts65Dn OPCs likewise exhibited increased expression of several genes whose functions have been associated with immune activation, such as *Adam10*, an extracellular matrix (ECM) protein involved in inflammatory signaling (Pabois et al., 2014) and *Ifngr2*, an IFN receptor, whose upregulation induces increased immunomodulatory signaling (Schroder et al., 2004). Trisomic astrocytes, microglia, and OPCs also showed increased expression of several proteasome subunits including *Psmb1/7* (Tanaka, 2009), as well as antioxidant enzymes *Prdx1/5*, whose functions are important during periods of oxidative stress (Park et al., 2016), and *Lamtor1/2*, which mediate cellular autophagy and survival responses to inflammation (Scheffler et al., 2014). Taken together, these transcriptional changes are consistent with previous reports of glial reactivity in the DS brain (Jørgensen et al., 1990; C. Chen et al., 2014; Pinto et al., 2020; Ponroy Bally and Murai, 2021).

### Cortical Ts65Dn OPCs exhibit enrichment for a senescence-associated gene signature and an altered cell surfaceosome landscape

**Figure 4.**
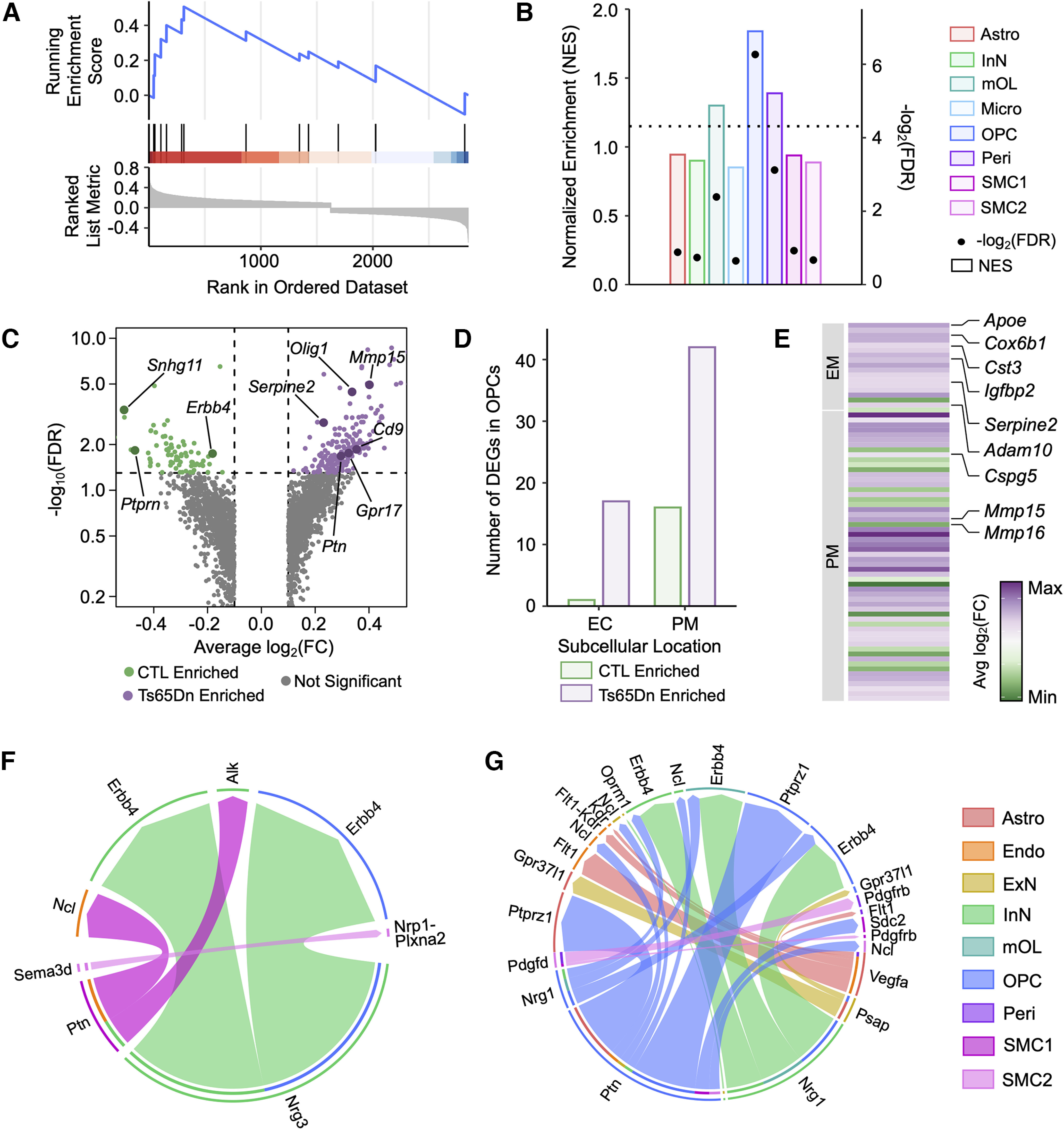
Cortical Ts65Dn OPCs exhibit a selective senescence-associated phenotype. ***A***, GSEA-based enrichment plot of the SenMayo senescence gene set for OPCs. The upper *y*-axis represents the enrichment score (ES), and the blue line represents the running enrichment score. The *x*-axis displays gene ranked according to their expression in Ts65Dn, with the most upregulated genes on the left-hand side and the most downregulated genes toward the right-hand side. Black vertical lines depict the positions of individual genes and their enrichment within the transcriptional signature. ***B***, Barplot displaying the enrichment of the SenMayo senescence gene set across cortical cell types. The bars correspond to the left *y*-axis, displaying normalized enrichment score for each cell type by pathway and are colored by cell type identity. The dots correspond to the right *y*-axis, displaying -log_2_(FDR) for each cell type by pathway; the dashed horizontal line intercepts the right *y*-axis at 4.3, corresponding to an FDR = 0.05. All dots above this horizontal line are statistically significant. ***C***, Volcano plot of OPC DEGs in Ts65Dn versus CTL conditions. Only genes with an average log_2_fold change [avg log_2_(FC)] >0.1 or <−0.1. are included in the plot. Each dot represents a gene. Dots are colored according to enrichment: green dots are enriched in CTL, purple dots are enriched in Ts65Dn, and gray dots are not significantly enriched in either condition. The horizontal line depicts an FDR = 0.05, such that all genes above this line are statistically significant. ***D***, Barplot depicting the number of DEGs (FDR < 0.05) in OPCs with functionality localized to the extracellular compartment (EC) or plasma membrane (PM). Purple bars depict DEGs enriched in Ts65Dn, and green bars depict DEGs enriched in CTL. ***E***, Heatmap of differentially expressed genes (DEGs) localized to the extracellular compartment (EC) or plasma membrane (PM) in OPCs. Each row represents a gene. The color code represents the avg log_2_(FC) of gene expression between Ts65Dn and CTL. ***F***, Chord diagram of cell-cell signaling pathways that are downregulated in Ts65Dn versus CTL. Cell type identity of the ligand is indicated in the outermost edge of the diagram, while the cell identity of the receptor is indicated by the internal ring. Colored arrows indicate the specific ligand-receptor (LR) pairs and are colored according to the outgoing ligand signal. ***G***, As in ***F***, but depicting cell-cell signaling pathways that are upregulated in Ts65Dn versus CTL.

Given reports of increased DNA damage and chronic neuroinflammation in DS and the Ts65Dn brain (Guedj et al., 2016; Ahmed et al., 2021; Puente-Bedia et al., 2022), we hypothesized that subpopulations of Ts65Dn cells would exhibit accelerated senescence. Senescent cells accumulate in aged tissues because of exhaustion of proliferation-competent cells (Di Micco et al., 2021; Kumari and Jat, 2021). However, the identification and characterization of senescent cells, particularly in bulk or single-nucleus sequencing data, is challenging because of the low average expression of many senescence-associated transcripts and the imprecise genetic definition of cellular senescence. We thus performed GSEA using a recently-published senescence-associated gene signature: SenMayo (Saul et al., 2022; Materials and Methods). This gene set is composed of 125 senescence-associated genes, including key SASP factors, transmembrane, and intracellular protein-encoding transcripts that have been validated across murine and human age-related transcriptomic datasets. Through this analysis, we observed selective enrichment (normalized enrichment score, NES > 0, FDR < 0.05) of the SenMayo gene set only in Ts65Dn OPCs (Fig. 4*A*,*B*), suggesting a cell type-specific senescence phenotype in this population.

Trisomic OPCs also demonstrated altered expression of several genes pertaining to maturation, proliferation, and inflammation (Fig. 4*C*). Among Ts65Dn-enriched OPC DEGs, we observed increased expression of *Gpr17*, which causes myelinogenesis defects when overexpressed in OL-lineage cells (Y. Chen et al., 2009; Ou et al., 2016), *Mif*, a proinflammatory cytokine that regulates NF-κB and p53 expression (Salminen and Kaarniranta, 2011; Kim et al., 2017), as well as *Mmp15* and *Adamts17*, which are endoproteases that mediate the activity and bioavailability of inflammatory factors, such as TNFα and IL-1 (Rosenberg, 2002; Cabral-Pacheco et al., 2020). GSEA analysis of Ts65Dn OPCs also revealed an enrichment for metabolic regulation by p53, a key TF whose upregulation induces growth arrest, apoptosis, and cellular senescence (Mijit et al., 2020).

Cellular senescence is also marked by the widescale disruption of protein secretion and expression, which leads to the acquisition of a senescence-associated secretory phenotype (SASP). The SASP includes chemokines, cytokines, matrix metalloproteinases (MMPs), interleukins, and proteases, which damage and modify the surrounding microenvironment (Coppé et al., 2010). Further examination of OPC DEGs revealed that Ts65Dn-upregulated DEGs include several SASP family members, including *Apoe*, *Igfbp2*, *Cst3*, *Serpine2*, and *Cox6b1* (Fig. 4*D*,*E*). Aberrant apolipoprotein E (APOE) accumulation has been shown to drive senescence through degradation of nuclear envelope proteins that lead to heterochromatin destabilization and disorganization (Zhao et al., 2022). IGFBP2 similarly induces and sustains cellular senescence by inhibiting apoptosis via the interaction with and protection of p21 (Mercurio et al., 2020). Alongside the induction of tissue sensing programs and secreted mediators, senescent cells demonstrate remodeling of the cell-surfaceome landscape through the upregulation of transcripts encoding plasma membrane (PM) proteins (H.A. Chen et al., 2023). Indeed, many of the upregulated DEGs in Ts65Dn OPCs encoded PM proteins, such as ECM molecules (*Adam10*, *Cspg5*, *Mmp15/16*), and VEGF signaling molecules (*Cadm4*, *Cd63*; Fig. 4*D*,*E*). Taken together, the increase in extracellular (EC) and PM-encoding transcripts suggests an enhanced capability to sense and secrete environmental cues permitting senescent Ts65Dn OPCs an increased interaction with and influence of their surrounding microenvironment.

### Ligand-receptor (LR) analysis of the Ts65Dn cortex identifies changes in intercellular communication

Given the importance of cell-cell signaling in shaping neural circuits, we performed ligand-receptor (LR) mapping in the six-month Ts65Dn and euploid CTL cortex using CellChat (S. Jin et al., 2021; Materials and Methods). We sought to build a comprehensive intercellular signaling network and better understand how trisomy-driven changes in gene expression affect cell-cell signaling by leveraging the transcriptional profiles of each cell population. Network analysis showed that 24 signaling pathways in the cortex were perturbed in Ts65Dn mice. Pleiotrophin (*Ptn*) and neuregulin (*Nrg*) were among the top mediators of disrupted crosstalk between various major cell types in the Ts65Dn cortex (Fig. 4*F*,*G*).

PTN is a ligand for several receptors in the brain, including protein tyrosine phosphatase receptor type Z1 (PTPRZ1), anaplastic lymphoma kinase (ALK), and nucleolin (*Ncl*; X. Wang, 2020; Papadimitriou et al., 2022). We found increased *Ptn* to *Ptprz1* signaling in OPCs, decreased *Ptn* to *Alk* signaling between SMC1 and InNs, and decreased *Ptn* to *Ncl* signaling between SMC1 and endothelial cells in the trisomic mouse brain (Fig. 4*F*,*G*). Upregulated PTN-PTPRZ1 signaling indirectly increases the bioavailability of β-catenin, whose activity can hinder both developmental myelination and adult remyelination (Harroch et al., 2002; McClain et al., 2012). The increased PTPRZ1 signaling in OPCs may thus contribute to the defective myelinogenesis observed in trisomy. Homeostatic PTN-ALK signaling promotes differentiation, growth, and survival (Bowden et al., 2002). This LR pair may therefore represent a key signaling node whose downregulation contributes to abnormalities and disruptions in Ts65Dn InN network activity (Huo et al., 2018; Giffin-Rao et al., 2022; Zorrilla de San Martin et al., 2020). NCL is expressed in endothelial cells and plays a role in angiogenesis and vascularization (Koutsioumpa et al., 2012). The downregulated signaling of a proangiogenic LR interaction in endothelial cells is thus consistent with prior observations of impaired endothelial vascular recruitment and mobilization in DS (Reynolds et al., 2010; Koutsioumpa et al., 2012).

Another set of perturbed pathways detected in Ts65Dn mice involved inferred interactions between Erbb2 receptor tyrosine kinase 4 (*Erbb4*) with neuregulin1 (*Nrg1*) and neuregulin3 (*Nrg3*). We uncovered increased *Nrg1* to *Erbb4* signaling in OPCs and InNs, and decreased *Nrg3* to *Erbb4* signaling between InNs and OPCs in the Ts65Dn cortex (Fig. 4*F*,*G*). NRG1 regulates neuronal plasticity and migration, OL-lineage maturation, myelination, and dendritic arborization (Mei and Xiong, 2008). In InNs, NRG1 signaling through ERBB4 plays a critical role in circuit development, neuronal differentiation and GABAergic transmission (Ting et al., 2011; Cahill et al., 2012). Similarly, this signaling is vital in regulating OPC growth, proliferation, and differentiation into mOLs (Brinkmann et al., 2008). The dysregulation of this LR interaction between InNs and OPCs may thus contribute to DS cognitive disability attributable to excitatory/inhibitory (E/I) imbalance (Souchet et al., 2014), modified neural-network excitability (Contestabile et al., 2017), and reduced axon myelination (Jose Luis Olmos-Serrano et al., 2016; Reiche et al., 2019). Like *Nrg1*, *Nrg3* is part of the neuregulin (*Nrg*) family and plays an important role in neural circuitry development through *Erbb4* signaling (D. Zhang et al., 1997; Mei and Nave, 2014). Its downregulation has been implicated in several neurologic and psychiatric conditions such as schizophrenia (Avramopoulos, 2018). Importantly, the imbalance of NRG signaling in the brain can drive neuropathology by compromising overall neural connectivity and circuit homeostasis. Thus, the disrupted reciprocal interaction we observe in the Ts65Dn *Nrg1/3* LR networks may promote broad disruptions in brain architecture.

### Ts65Dn trisomy induces unique chromatin accessibility patterns in the mature cortex

**Figure 5.**
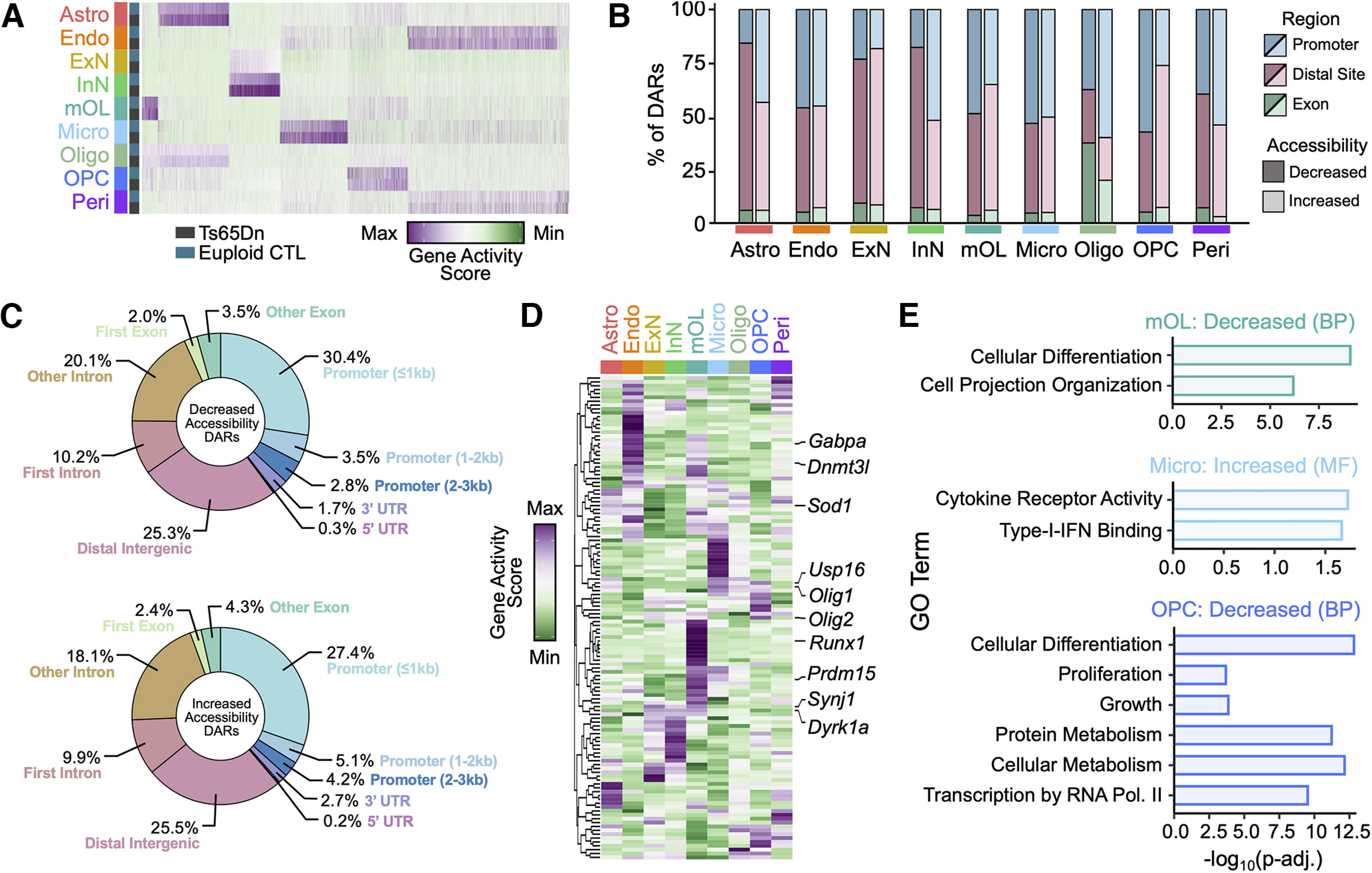
Chromatin accessibility landscape in the mature Ts65Dn cortex. ***A***, Heatmap of the average number of cut sites within a DAR for each cell type by condition. Each column represents a cell type from Ts65Dn or CTL. The color code represents the gene activity score. ***B***, Stacked barplot displaying the genomic distribution of differentially accessible regions (DARs). DARs located within promoters (<0–3 kb of the gene) are colored in blue, within distal regulatory sites (5′/3′ UTRs, introns, >300 kb downstream of the gene or intergenic) are colored in pink, or within exons are colored in green. Bars shaded in darker colors correspond to DARs showing decreased accessibility in Ts65Dn, and bars shaded in lighted colors correspond to DARs showing increased accessibility in Ts65Dn. Bars are grouped according to cell type identity. ***C***, Pie chart of the genomic distribution of DARs that exhibit decreased or increased accessibility in Ts65Dn mice. Each fraction of the pie corresponds to a different genomic region, and is labeled according to the percentage of DARs associated with the specific genomic region. ***D***, Hierarchically clustered heatmap of normalized gene accessibility for genes contained within the Ts65Dn chromosomal product. The plot displays gene activity across all cell types in snATAC-seq. Several genes of interest are indicated. Each column represents a cell type. The color code represents the row-normalized accessibility for each gene. ***E***, Barplot of the -log_10_(p-adj) value of enrichment for select gene ontology (GO) biological process (BP) or molecular function (MF) terms for mature oligodendrocytes (mOL), microglia (Micro), and oligodendrocyte precursor cells (OPC). Bars are colored according to cell type.

To identify cell type-specific changes in the chromatin landscape of the Ts65Dn cortex, we analyzed differentially accessible regions (DARs) in snATAC-seq (Materials and Methods). We used DARs that met an FDR value of 0.05 to define statistically significant changes in chromatin-level expression between Ts65Dn and CTL offspring. We found that DARs were highly heterogeneous across cortical cell types (Fig. 5*A*; Table 4) and a majority (75.1%) were downregulated (Fig. 5*B*). To assess the genomic distribution of DARs, we used ChIPseeker (Q. Wang et al., 2022; Materials and Methods). We found that the distribution of DARs was relatively consistent across cell types, with a majority occurring in distal regulatory sites (Fig. 5*B*). Across DARs, ∼6% were found in exons, ∼36% were found in promoters (<0–3 kb), and ∼56% were found in distal regulatory sites (5′/3′ UTR, introns, >300kb downstream, or intergenic; Fig. 5*C*).

**Table 4:** Top five loci with increased accessibility in each cortical cell type in Ts65Dn mice compared with CTL

Gene	FDR	log_2_(FC)	Cell type	Gene	FDR	log_2_(FC)	Cell type
*Acsl3*	1.24E-31	0.38	Astro	*Dscaml1*	7.58E-13	0.21	mOL
*Enthd1*	1.96E-14	0.31	Astro	*Prr18*	3.44E-11	0.21	mOL
*Tiam2*	1.63E-16	0.31	Astro	*Dleu2*	1.07E-10	0.50	Micro
*Gas7*	1.62E-12	0.30	Astro	*Magi1*	7.32E-08	0.42	Micro
*Ripk4*	7.13E-11	0.29	Astro	*Ccr6*	9.00E-07	0.33	Micro
*Mlph*	2.68E-10	0.56	Endo	*Ccr6*	9.37E-05	0.30	Micro
*Rnf150*	8.14E-10	0.54	Endo	*Chsy1*	2.92E-06	0.28	Micro
*Slc5a6*	8.37E-11	0.53	Endo	*Snx33*	2.22E-02	0.46	Oligo
*Calr3*	7.76E-15	0.51	Endo	*Cnbd2*	2.22E-02	0.43	Oligo
*Coro2b*	2.15E-12	0.47	Endo	*Dtna*	4.13E-02	0.30	Oligo
*Car10*	2.29E-144	0.25	ExN	*Dyrk1a*	1.84E-02	0.22	Oligo
*Dennd1b*	1.16E-121	0.24	ExN	*Rps6ka2*	2.22E-02	0.22	Oligo
*Ifi202b*	1.51E-66	0.17	ExN	*Itga9*	3.93E-06	0.34	OPC
*Dleu2*	5.09E-64	0.16	ExN	*Olig2*	3.58E-09	0.32	OPC
*Pde10a*	5.43E-51	0.15	ExN	*Ptpn14*	2.11E-05	0.31	OPC
*Cldn17*	1.44E-14	0.16	InN	*Cspg4*	4.11E-05	0.29	OPC
*Acoxl*	1.84E-13	0.16	InN	*Olig2*	7.81E-05	0.29	OPC
*Atp5j*	1.21E-41	0.16	InN	*Trp53i11*	1.18E-02	0.46	Peri
*Arhgap15*	1.09E-10	0.16	InN	*Msx1*	5.06E-03	0.44	Peri
*Mis18a*	1.25E-11	0.15	InN	*Rps2-ps6*	1.04E-02	0.43	Peri
*Fcho1*	1.52E-81	0.50	mOL	*Gltp*	6.28E-04	0.42	Peri
*Mylk*	1.78E-17	0.27	mOL	*Rps6ka2*	2.09E-03	0.41	Peri
*App*	8.82E-22	0.23	mOL				

Listed are the top five genes [ranked by highest average log_2_(FC)] that show significant differential accessibility in Ts65Dn mice compared with CTL as detected via snATAC-seq. Shown are fold-change values of the average expression for each gene and the false discovery rate (FDR) between Ts65Dn and CTL cells. Each cell type is listed in the final column and is abbreviated as described in Table 1.

Ts65Dn cells exhibited increased accessibility of ∼25% of Ts65Dn-encoded genes at the chromatin level. Similar to snRNA-seq, we observed heterogeneous accessibility levels of Ts65Dn triplicated genes across cell types, with only a few gene loci including the antioxidant *Sod1*, the kinase *Dyrk1a*, and the spliceosome component *Son* at significantly higher levels in at least seven out of nine identified cortical cell types (Fig. 5*D*). Increased chromatin accessibility of *Olig1* and *Olig2* loci was observed selectively in astrocytes, mOLs, and OPCs. Overexpression of *Olig2* has been shown to preferentially drive progenitors toward a GFAP+ reactive astrocyte fate rather than an OL-lineage fate (Lu et al., 2012) and can trigger transcriptional repression during myelinogenesis (K. Zhang et al., 2022). The *Runx1* locus exhibited increased accessibility in Ts65Dn microglia; RUNX1 is a TF that modulates microglial gene expression during early postnatal life, but can increase in adults following brain injury or infection to promote microglial activation (Zusso et al., 2012; Yeh and Ikezu, 2019). *Synj1* was another locus that showed selective increased accessibility in trisomic InNs and is known for its involvement with endocytosis and synaptic vesicle cycling (Choudhry et al., 2021). Specifically, elevated levels of SYNJ1 have been shown to trigger deficits in age-dependent long-term memory retention in individuals with DS-related AD (Miranda et al., 2018), thus implicating SYNJ1 as a key mediator of cognitive disability.

We next employed gene ontology (GO) analysis to assess biological pathways associated with chromatin accessibility changes in Ts65Dn mice (Materials and Methods; Table 5; Raudvere et al., 2019). Among downregulated DARs from trisomic OPCs and mOLs, we observed an enrichment of GO categories associated with cell differentiation, projection organization, and regulation of metabolic processes (Fig. 5*E*). We also found enrichment of biological process (BP) ontologies associated with growth and proliferation in trisomic OPC DARs with decreased accessibility, further suggesting a disruption in OPC development and a growth arrest typical of a senescence phenotype (Fig. 5*E*). Consistent with the hyperactive inflammatory milieu known to occur in the trisomic brain (Wilcock, 2012; Flores-Aguilar et al., 2020), we observed an enrichment of cytokine receptor activity and type-I-IFN binding among the top five enriched molecular function (MF) categories for increased accessibility DARs in Ts65Dn microglia (Fig. 5*E*). Taken together, these findings demonstrate that trisomy has a profound genome-wide impact on gene expression, with a notable effect on genes related to inflammation and differentiation in glial cell populations.

**Table 5:** Top three Ts65Dn enriched biological processes (BP) in each cortical cell type identified via gene ontology (GO) analysis in Ts65Dn mice compared with CTL

GO biological process (BP) pathway	FDR	Gene ratio	Cell type
System development	1.05E-05	0.35	Astro
Positive regulation of biological process	1.76E-05	0.45	Astro
Regulation of metabolic process	7.97E-05	0.47	Astro
Anatomical structure development	1.66E-20	0.42	Endo
Negative regulation of cellular process	1.57E-19	0.36	Endo
Developmental process	3.78E-19	0.44	Endo
Phosphate-containing compound metabolic process	6.34E-04	0.40	ExN
Phosphorus metabolic process	7.09E-04	0.40	ExN
Organophosphate metabolic process	4.31E-03	0.24	ExN
Synapse assembly	6.34E-03	0.10	InN
Animal organ development	1.27E-02	0.37	InN
Positive regulation of gene expression	1.92E-02	0.20	InN
Cell development	1.08E-07	0.32	mOL
Neurogenesis	3.24E-07	0.27	mOL
Nervous system development	9.38E-07	0.31	mOL
Regulation of cellular process	2.93E-05	0.67	Micro
Biosynthetic process	3.88E-05	0.41	Micro
Cellular biosynthetic process	7.17E-05	0.40	Micro
Multicellular organism development	5.54E-22	0.44	OPC
Anatomical structure development	3.08E-21	0.49	OPC
System development	1.13E-19	0.40	OPC
Cellular localization	2.48E-05	0.35	Peri
Phosphate-containing compound metabolic process	7.90E-04	0.29	Peri
Phosphorus metabolic process	9.26E-04	0.29	Peri

Listed are the top three biological processes (BP) identified via GO analysis (ranked by lowest FDR value) that are significantly enriched in Ts65Dn mice compared with CTL. The gene ratio is defined as the ratio between the intersection size and query size. Oligo cells were not included in this list, as no pathways were significantly enriched (FDR < 0.05) between conditions. Each cell type is listed in the final column and is abbreviated as described in Table 1.

### Ts65Dn OPCs exhibit an altered chromatin state consistent with cellular senescence

**Figure 6.**
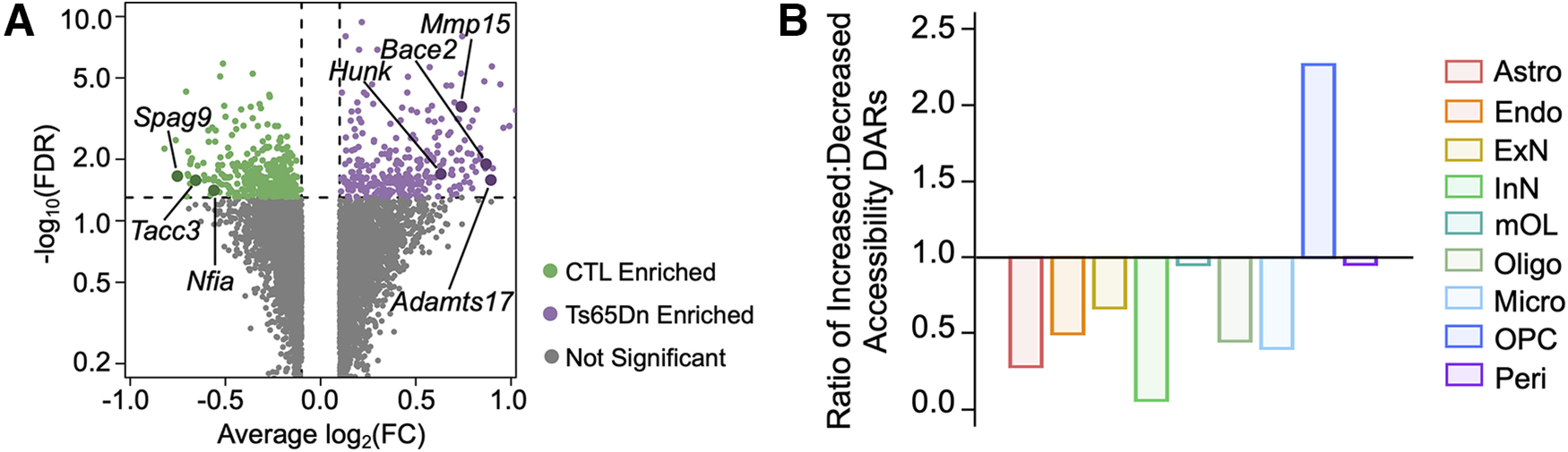
Cortical Ts65Dn OPCs exhibit senescence-associated chromatin changes. ***A***, Volcano plot of OPC DARs in Ts65Dn versus CTL conditions. Only genes with an average log_2_fold change [log_2_(FC)] >0.1 or <−0.1. are included in the plot. Each dot represents a gene. Dots are colored according to enrichment: green dots are enriched in CTL, purple dots are enriched in Ts65Dn, and gray dots are not significantly enriched in either condition. The horizontal line depicts an FDR = 0.05, such that all genes above this line are statistically significant. ***B***, Barplot displaying the ratio of DARs with increased to decreased accessibility across all cell types. A ratio of 1 indicates an equal number of increased and decreased DARs, while a ratio of greater or <1 indicates a greater number of increased or decreased DARs, respectively. Each bar is colored by cell type identity. Only statistically significant DARs (FDR < 0.05) are included.

To assess locus-specific changes in Ts65Dn OPC chromatin accessibility, we more closely examined population-level DARs. Herein, we observed reduced accessibility at several cell growth and mitosis regulatory loci (Fig. 6*A*), including *Tacc3*, *Knstrn*, *Ptprg*, and *Spag9*, whose suppression can induce cell-cycle arrest and tend cells toward a senescent state (C.S. Lee et al., 2014; Cheung et al., 2015; Suhail et al., 2015; Jagadish et al., 2016). Further, we observed increased accessibility at several chondroitin sulfate proteoglycans (CSPG) loci, including *Tnr*, *Bcan*, *Vcan*, *Acan*, and *Ncan* (Jang et al., 2020; Fig. 6*A*). CSPGs have been found to negatively regulate OPC proliferation, differentiation and remyelination, as well as induce production of proinflammatory cytokines (Ghorbani and Yong, 2021; Tewari et al., 2022). Similar to our findings in snRNA-seq data, we found that Ts65Dn OPCs exhibited an increase in accessibility at several endoprotease loci, including *Mmp15/19*, *Adam12*, and *Adamts5/17* loci (Fig. 6*A*), all of which play a role in modulating neuroimmune activity through the regulation of proinflammatory cytokine availability and signaling (Rosenberg, 2002; Cabral-Pacheco et al., 2020).

Structural changes within and around senescent cells are also accompanied by dramatic molecular changes to the epigenetic landscape, including a global reduction in heterochromatin (Sadaie et al., 2013; Nacarelli et al., 2017; Sidler et al., 2017). To investigate whether cortical Ts65Dn OPCs exhibited this senescence-associated loss of heterochromatin, we compared the number of DARs with increased or decreased accessibility in Ts65Dn versus euploid CTL across all cell types. We found a greater number of peaks with increased accessibility in Ts65Dn relative to CTL only in OPCs, consistent with a net increase in chromatin accessibility (Fig. 6*B*). All other cell types profiled through snATAC-seq exhibited a greater number of peaks with decreased accessibility, suggestive of a cell type-specific loss of heterochromatin consistent with selective senescence in Ts65Dn OPCs.

### Cell type-specific transcription factors (TF) in the trisomic mouse cortex

**Figure 7.**
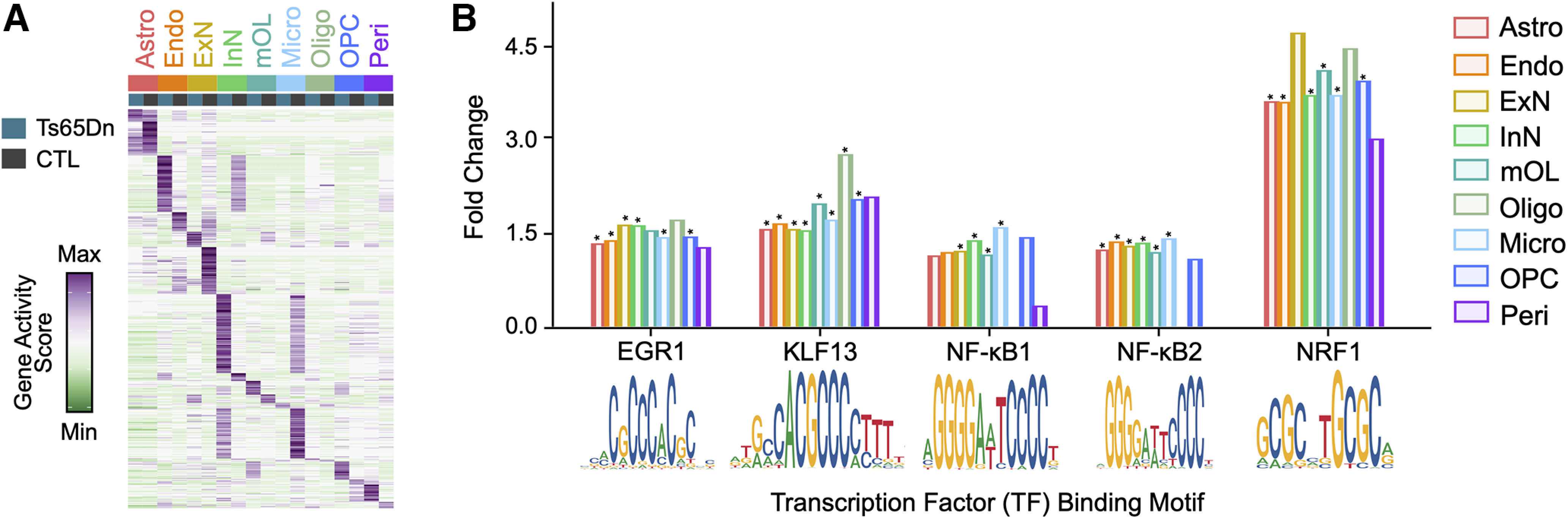
Cell type-specific transcription factor (TF) activity in the mature Ts65Dn cortex. ***A***, Heatmap of average ChromVAR transcription factor (TF) motif activity for each cell type. Each column represents a cell type from Ts65Dn or CTL. The color code represents the row-normalized accessibility for each TF. ***B***, Barplot showing the relative fold change for select TFs between Ts65Dn and CTL. Each bar corresponds to a cell type. Bars labeled with asterisks indicate statistically significant enrichment (FDR < 0.05) for the given TF. Beneath each grouped barplot is the position weight matrix (PWM) for the binding motif associated with each TF.

To gain insight into transcription factor (TF)-mediated gene regulation in Ts65Dn mice, we constructed cell type-specific TF regulatory networks based on the presence of binding motifs within DARs. We used chromVAR (Schep et al., 2017) to identify shared and cell type-specific gene-chromatin interactions using our snATAC-seq data and observed unique TFs enriched across all cortical cell types (Materials and Methods; Fig. 7*A*; Table 6).

**Table 6:** Top five transcription factor (TF) binding motifs enriched within DARs in each cortical cell type in Ts65Dn mice compared with CTL

Motif	FDR	FE	Cell type	Motif	FDR	FE	Cell type
HINFP	4.40E-116	4.34	Astro	HINFP	2.89E-308	4.71	mOL
ZBTB33	1.29E-74	4.17	Astro	ZBTB33	1.29E-167	4.50	mOL
ZBTB14	3.16E-232	3.69	Astro	YY2	1.03E-263	3.84	mOL
NRF1	4.23E-241	3.63	Astro	ELK1	9.27E-205	3.48	mOL
TFDP1	1.84E-164	3.62	Astro	HES1	5.44E-200	3.21	mOL
HINFP	7.04E-59	4.21	Endo	HINFP	7.84E-32	4.09	Micro
ZBTB14	7.86E-141	3.83	Endo	TFDP1	2.05E-58	3.84	Micro
NRF1	4.69E-134	3.62	Endo	NRF1	2.24E-76	3.73	Micro
ZBTB33	1.05E-25	3.44	Endo	YY2	1.95E-34	3.69	Micro
TFDP1	4.72E-81	3.41	Endo	ZBTB14	1.76E-66	3.62	Micro
ZFP57	3.33E-240	2.44	ExN	KLF14	1.64E-02	5.79	Oligo
E2F2	1.52E-62	2.27	ExN	NRF1	2.88E-43	3.96	OPC
CENPB	2.12E-88	2.27	ExN	ZBTB14	3.04E-37	3.82	OPC
E2F1	1.29E-66	2.23	ExN	HINFP	3.74E-13	3.81	OPC
E2F4	3.49E-65	2.20	ExN	ZBTB33	2.17E-07	3.54	OPC
ZBTB33	5.01E-238	4.13	InN	TCFL5	3.67E-19	3.50	OPC
HES1	2.82E-248	2.88	InN	E2F1	1.58E-06	17.31	Peri
ELK1	4.88E-178	2.70	InN	KLF15	1.21E-02	2.72	Peri
YY2	3.61E-179	2.69	InN	KLF14	3.52E-02	2.54	Peri
EGR2	6.27E-254	2.30	InN				

Listed are the top five TF motifs [ranked by highest fold enrichment (FE)] that show significant enrichment in Ts65Dn mice compared with CTL. Shown are FE values for each binding motif and the false discovery rate (FDR) between Ts65Dn and CTL cells. Each cell type is listed in the final column and is abbreviated as described in Table 1.

**Table 7:** Statistical table

	Figure	Data structure	Type of test	Power	*p*-value
1	8*B*’	Normal distribution	Two-tailed unpaired Student’s *t* test	6.79	*p *=* *0.021
2	8*B*’’	Normal distribution	One-way ANOVA with a *post hoc* Tukey	305.25	Ts65Dn vs CTL = 1.3 × 10^−6^ Ts65Dn+fisetin vs CTL = 0.054 Ts65Dn+fisetin vs Ts65Dn = 2.5 × 10^−6^
3	8*C*’	Normal distribution	Two-tailed unpaired Student’s *t* test	9.97	*p *=* *0.0021
4	8*C*’’	Normal distribution	One-way ANOVA with a *post hoc* Tukey	24.87	Ts65Dn vs CTL = 0.0063 Ts65Dn+fisetin vs CTL = 0.215 Ts65Dn+fisetin vs Ts65Dn = 0.0011
5	8*G*’	Normal distribution	Two-tailed unpaired Student’s *t* test	5.05	*p *=* *0.014
6	8*G*’’	Normal distribution	One-way ANOVA with a *post hoc* Tukey	15.86	Ts65Dn vs CTL = 0.0076 Ts65Dn+fisetin vs CTL = 0.963 Ts65Dn+fisetin vs Ts65Dn = 0.0059
7	8*H*’	Normal distribution	Two-tailed unpaired Student’s *t* test	14.13	*p *=* *0.0049
8	8*H*’’	Normal distribution	One-way ANOVA with a *post hoc* Tukey	24.84	Ts65Dn vs CTL = 0.0013 Ts65Dn+fisetin vs CTL = 0.936 Ts65Dn+fisetin vs Ts65Dn = 0.0019
9	9*B*’’	Normal distribution	One-way ANOVA with a *post hoc* Tukey	11.63	Ts65Dn vs CTL = 0.0032 Ts65Dn+fisetin vs CTL = 0.514 Ts65Dn+fisetin vs Ts65Dn = 0.017
10	8*J*’	Normal distribution	Two-tailed unpaired Student’s *t* test	8.68	*p *=* *0.013
11	8*J*’’	Normal distribution	One-way ANOVA with a *post hoc* Tukey	6.73	Ts65Dn vs CTL = 0.039 Ts65Dn+fisetin vs CTL = 0.976 Ts65Dn+fisetin vs Ts65Dn = 0.050
12	10*A*’	Normal distribution	Two-tailed unpaired Student’s *t* test	4.43	*p *=* *0.047
13	10*A*’’	Normal distribution	One-way ANOVA with a *post hoc* Tukey	8.64	Ts65Dn vs CTL = 0.018 Ts65Dn+fisetin vs CTL = 0.718 Ts65Dn+fisetin vs Ts65Dn = 0.046
14	10*B*’’	Normal distribution	One-way ANOVA with a *post hoc* Tukey	11.39	Ts65Dn vs CTL = 0.0091 Ts65Dn+fisetin vs CTL = 0.506 Ts65Dn+fisetin vs Ts65Dn = 0.033
15	7*E*	Normal distribution	One-way ANOVA with a *post hoc* Tukey	10.31	Ts65Dn vs CTL = 0.0016 Ts65Dn+fisetin vs CTL = 0.973 Ts65Dn+fisetin vs Ts65Dn = 0.0011
16	7*E*	Normal distribution	One-way ANOVA with a *post hoc* Tukey	11.68	Ts65Dn vs CTL = 0.00013 Ts65Dn+fisetin vs CTL = 0.0044 Ts65Dn+fisetin vs Ts65Dn = 0.294

ChromVAR detected enrichment of the early growth response factor 1 (EGR1) TF binding site in DS InNs (Fig. 7*B*), which was supported by increased *Egr1* mRNA in Ts65Dn InNs. EGR1 regulates the development of GABA receptor subunit genes, thus acting as a key regulator of InN development (Mo et al., 2015). KLF13, which binds to regulatory regions of genes that are important for OL differentiation and maturation, such as *Mag*, *Mbp*, and *Plp1* (Bernhardt et al., 2022), similarly showed increased motif accessibility and increased *Klf13* expression in snRNA-seq (Fig. 7*B*).

In trisomic endothelial cells, ExNs, InNs, microglia, and mOLs, we observed a marked increase in motif enrichment for nuclear factor κ-light-chain-enhancer of activated B cells (NF-κB1 and NF-κB2), a critical modulator of cell growth, cell survival, development, and inflammation (Liu et al., 2017; Fig. 7*B*). This increase in NF-κB1/2 TF accessibility across several cortical cell types suggests an increase in inflammatory signaling, consistent with the hyperimmune milieu observed in trisomy. Additionally, we found an enrichment of nuclear respiratory factor 1 (NRF1) motif accessibility in all trisomic major neuronal and non-neuronal cell types except for mOLs and pericytes (Fig. 7*B*). NRF1 is a key regulator of cell growth, apoptosis, senescence, neurogenesis, genomic instability, and mitochondrial function (Preciados et al., 2016). A disruption in mitochondrial biogenesis, driven by NRF1 dysregulation, has been observed in many neurodegenerative and neurodevelopmental disorders including AD, Parkinson’s disease (PD), and Huntington’s disease (HD), and may play a similar role in widespread neurogenesis or myelination deficits in DS (Satoh et al., 2013; Preciados et al., 2016; Morabito et al., 2021). Lastly, we noted enrichment of the hairy and enhancer of split-1 (HES1) motif in Ts65Dn ExNs, InNs, and OPCs relative to euploid CTL (Fig. 7*B*). HES1 represses proneural or differentiation genes to keep stem cell progenitors in quiescent or proliferative states (Ogata et al., 2011; Fan et al., 2018; Hu and Zou, 2022). Importantly, HES1 expression is downregulated during myelination, and HES1 overexpression in OPCs results in impaired oligodendrogenesis (Li et al., 2021). Together, these results show that Ts65Dn trisomy leads to widespread perturbation of TF activity and binding motif accessibility, which may contribute to key elements of trisomy-associated neuropathology.

### Deep layer cortical Ts65Dn OPCs display increased senescence

**Figure 8.**
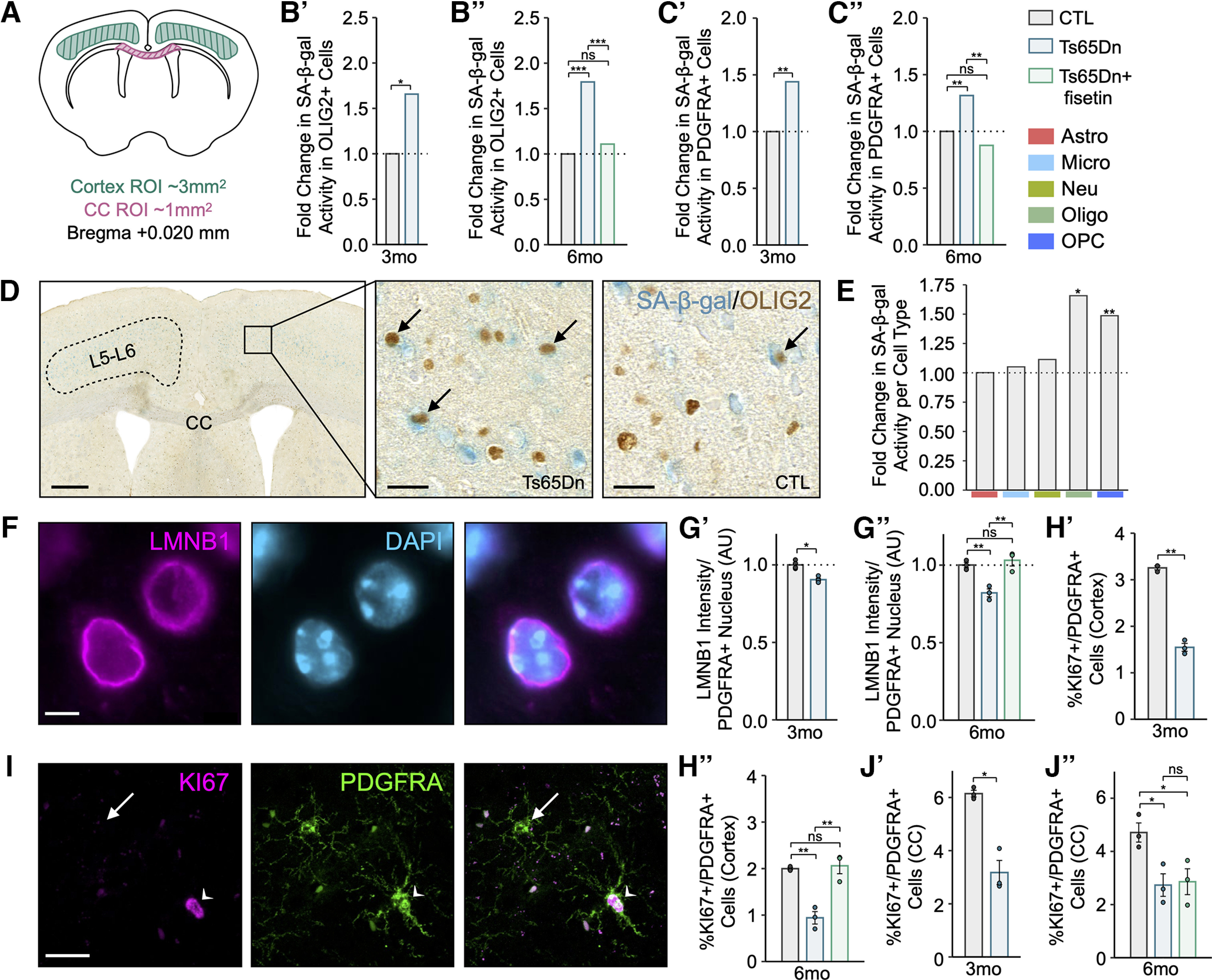
Deep cortical layer Ts65Dn OPCs exhibit several hallmarks of senescence that are rescued in mature adult mice treated with fisetin. ***A***, Schematic of the region of interest (ROI) used for cell quantification across the cortex (∼3 mm^2^) and CC (∼1 mm^2^), demarcated with a green and magenta border, respectively. All tissue was sliced at bregma +0.020 mm. ***B’***, ***B’’***, Barplot of SA-β-gal activity in OLIG2+ cells (OL-lineage) in three- and six-month mice, showing a significant increase in SA-β-gal+/OLIG2+ cells in Ts65Dn at both time points, with a rescue in six-month Ts65Dn mice treated with fisetin. Bars represent the average values for each condition (*n *=* *3 male mice per condition, *n *=* *3 replicates per mouse), and dots represent the average values for each mouse per condition. ***C’***, ***C’’***, As in ***B*** but depicting SA-β-gal activity in PDGFRA+ cells (OPCs), showing a significant increase in SA-β-gal+/PDGFRA+ cells in Ts65Dn at both time points, with a rescue in six-month Ts65Dn mice treated with fisetin (*n *=* *4 mice per condition, *n *=* *3 replicates per mouse, *n *=* *3 males, *n *=* *1 female). ***D***, SA-β-gal staining in coronal brain sections from a three-month Ts65Dn mouse; arrows point to OLIG2+ (brown) SA-β-gal+ (blue) cells throughout the cortex. Panel on the right shows a high magnification image of the area demarcated in the left panel. Images are representative of those observed in samples from Ts65Dn and CTL mice. Scale bars are shown in the bottom left corner of each panel: left panel, 500 μm; right/center panels, 50 μm. ***E***, Barplot of the fold change in SA-β-gal activity across several major cortical cell types in three-month Ts65Dn versus CTL mice (*n *=* *3 male mice per condition, *n *=* *3 replicates per mouse). Astro, astrocytes; Micro, microglia; Neu, neurons; Oligo, oligodendrocytes; OPC, oligodendrocyte precursor cells. Only Oligos and OPCs exhibit statistically significant increases in SA-β-gal activity in Ts65Dn versus CTL. ***F***, Immunostaining for LMNB1 (pink) and nuclear DAPI (blue) in a three-month Ts65Dn mouse. Images are representative of those observed in samples from Ts65Dn and CTL mice. Scale bar is shown in the bottom left corner of the panel: 5 μm. ***G’***, ***G’’***, Barplots of LaminB1 (LMNB1) intensity in arbitrary units (AU) per OPC (PDGFRA+) nucleus. Values have been normalized to the mean intensity found in CTL replicates. Bars represent the average values for each condition (at 3 months: *n *=* *4 mice per condition, *n *=* *3 replicates per mouse, *n *=* *3 males and *n *=* *1 female; at 6 months: *n *=* *3 male mice per condition, *n *=* *3 replicates per mouse), and dots represent the average values for mouse per condition. ***H’***, ***H’’***, Barplots of the percentage of KI67+/PDGFRA+ cells (proliferating OPCs), showing a significant increase in proliferating OPCs cells in Ts65Dn at both time points, with a rescue in six-month Ts65Dn mice treated with fisetin. Bars represent the average values for each condition (*n *=* *3 male mice per condition, *n *=* *3 replicates per mouse), and dots represent the average values for each mouse per condition. ***I***, Immunostaining for KI67 (pink) and PDGFRA (green) in a three-month Ts65Dn mouse. Images are representative of those observed in samples from Ts65Dn and CTL mice (*n *=* *3 male mice per condition, *n *=* *3 replicates per mouse). The arrow points to a PDGFRA+ cell, and the arrowhead points to a KI67+/PDGFRA+ cell. Scale bar is shown in the bottom left corner of the panel: 20 μm. ***J’***, ***J’’***, Barplots of the percentage of KI67+/PDGFRA+ cells (proliferating OPCs), showing a significant increase in proliferating OPCs cells in Ts65Dn at both time points, with a rescue in six-month Ts65Dn mice treated with fisetin. Bars represent the average values for each condition (*n *=* *3 male mice per condition, *n *=* *3 replicates per mouse), and dots represent the average values for each mouse per condition. For all plots: significance is determined using the two-tailed Student’s *t* test at three months, or using the one-way ANOVA and *post hoc* Tukey’s test at six months. *p-value < 0.05, **p-value < 0.01, ***p-value < 0.001, ns: not significant; error bars represent the average ± 1 standard deviation (SD).

To support our finding of an enriched senescence-associated gene signature in Ts65Dn OPCs, we assayed coronal brain sections from three- and six-month mice for lysosomal senescence-associated-β-galactosidase (SA-β-gal) activity coupled with immunohistochemistry (IHC; Materials and Methods). We profiled several major cortical cell types using antibodies against canonical marker genes, including astrocytes (AQP4+), microglia (IBA1+), neurons (NEUN+), OL-lineage cells (OLIG2+), and OPCs (PDGFRA+). Analysis of the cortex was restricted to a region of interest (ROI) of ∼3 mm^2^ across the motor and somatosensory regions (Fig. 8*A*; Materials and Methods). Ts65Dn mice exhibited a 1.7-fold increase in OLIG2+ SA-β-gal activity at three months (*t*_(1)_ = 6.79, *p* = 0.021; Table 7) and a 1.8-fold increase in OLIG2+ SA-β-gal activity at six months (Genotype effect, *F*_(2)_ = 305.25, *p* = 1.3 × 10^−6^), as well as a 1.4-fold increase in PDGFRA+ SA-β-gal activity at three months (*t*_(3)_ = 9.97, *p* = 0.0021) and a 1.3-fold increase in PDGFRA+ SA-β-gal activity at six months (Genotype effect, *F*_(4)_ = 24.87, *p* = 0.0063; Fig. 8*B*,*C*). Intriguingly, we observed that the increase in OL-lineage senescence was restricted to the deep layers (L5–L6) of the Ts65Dn cortex and was absent in the upper cortical layers (L1–L4) and the corpus callosum (CC; Fig. 8*D*). No other major cortical cell type demonstrated a significant increase in SA-β-gal activity in Ts65Dn mice (Fig. 8*E*). Overall, this analysis is suggestive of a regional and cell type-specific senescence phenotype.

To confirm these findings, we analyzed three- and six-month coronal sections for additional hallmarks of senescent cells: reduction of nuclear envelope protein LaminB1 (LMNB1) and reduction of proliferation (Freund et al., 2012; Kumari and Jat, 2021). LMNB1 decline associated with senescence can be detected at the protein-level through immunofluorescence (IF) staining and subsequent measurement of fluorescence intensity. We thus stained for LMNB1 in Ts65Dn and euploid CTL tissues in a cell type-specific manner (using PDGFRA to stain for OPCs) and compared LMNB1 fluorescence intensity between conditions (Materials and Methods). As expected, we observed a decline in cortical OPC (PDGFRA+) LMNB1 intensity by 10.4% at three months (*t*_(5)_ = 5.05, *p* = 0.014) and 14.4% at six months (Genotype effect, *F*_(6)_ = 15.86, *p* = 0.0076; Fig. 8*F–H*). Senescent cells are also typified by an irreversible cell cycle and proliferation arrest (Kumari and Jat, 2021). KI67 is only detected in actively dividing and not quiescent or senescent cells. Proliferating cell populations can thus be identified by quantifying nuclear KI67 positivity. We observed a 51.7% and 58.0% reduction in proliferating KI67+ OPCs in the Ts65Dn cortex at three months (*t*_(7)_ = 14.13, *p* = 0.049) and six months (Genotype effect, *F*_(8)_ = 24.84, *p* = 0.0013), respectively (Fig. 8*I*,*J*), suggestive of a growth arrest typical of senescence in trisomic OPCs.

### OPC senescence alters progenitor abundance but not oligodendrocyte maturation or myelination in the Ts65Dn cortex

**Figure 9.**
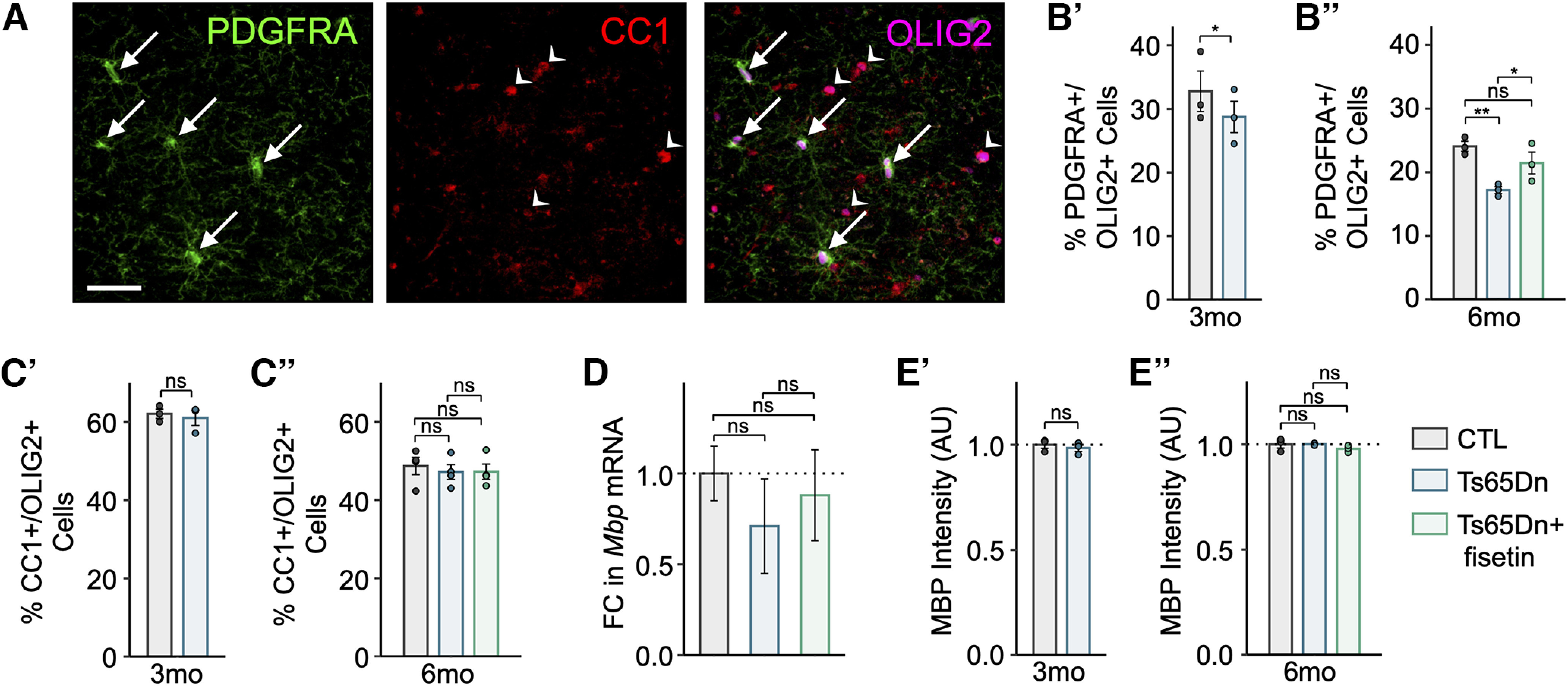
OPC senescence alters the number of progenitors but not the number of mature OLs. ***A***, Immunostaining for PDGFRA (green), CC1 (red), and OLIG2 (pink) in a three-month Ts65Dn mouse. Images are representative of those observed in samples from Ts65Dn and CTL mice. Arrows point to PDGFRA+/OLIG2+ cells, and arrowheads point to CC1+/OLIG2+ cells. Scale bar is shown in the bottom left corner of the panel: 20 μm. ***B’***, ***B’’***, Barplots of the percentage of PDGFRA+/OLIG2+ cells (OPCs), showing a significant decrease in the number of OPCs at six months, with a rescue in six-month Ts65Dn mice treated with fisetin. Bars represent the average values for each condition (at 3 months: *n *=* *3 male mice per condition, *n *=* *3 replicates per mouse; at 6 months: *n *=* *3 male mice per condition, *n *=* *3 replicates per mouse), and dots represent the average values for each mouse per condition. ***C’***, ***C’’***, As in ***B*** but depicting the percentage of CC1+/OLIG2+ cells (mOLs), showing no significant change in the number of cells in Ts65Dn at either time point, and no rescue in six-month Ts65Dn mice treated with fisetin (at 3 months: *n *=* *3 male mice per condition, *n *=* *3 replicates per mouse; at 6 months: *n *=* *4 mice per condition, *n *=* *3 replicates per mouse, *n *=* *3 males and *n *=* *1 female). ***D***, Barplot of the fold change (FC) in *Mbp* mRNA as measured through quantitative PCR (qPCR). Values were first normalized to *Gapdh*, and then normalized to the mean expression found in CTL replicates. Bars represent the average values for each condition (*n *=* *3 mice per condition, *n *=* *4 replicates per mouse, *n *=* *3 males). ***E’***, ***E’’***, As in ***B***, but depicting MBP intensity in arbitrary units (AU). Values have been normalized to the mean intensity found in CTL replicates (*n *=* *3 male mice per condition, *n *=* *3 replicates per mouse). For all plots: significance is determined using the two-tailed Student’s *t* test at three months, or using the one-way ANOVA and *post hoc* Tukey’s test at six months. *p-value < 0.05, **p-value < 0.01, ***p-value < 0.001, ns: not significant; error bars represent the average ± 1 standard deviation (SD).

To determine how OPC senescence affects cortical populations of OL-lineage cells, we quantified changes in OPC and mOL cell counts in coronal brain sections from Ts65Dn and euploid CTL mice. Prior studies have shown that trisomy-associated triplication of OLIG2 results in a transient early-life increase in OPCs, followed by a progressive decline in the progenitor pool with a resulting deficit in myelination that persists into adulthood (Lu et al., 2012; Jose Luis Olmos-Serrano et al., 2016). We stained for OLIG2 to mark the OL-lineage, and PDGFRA or CC1 to identify OPCs or mOLs, respectively. At both the three- and six-month time points, we discovered a decline in the percentage of PDGFRA+/OLIG2+ cells in the cortex, but only that at the six-month time point reached statistical significance (Genotype effect, *F*_(9)_ = 11.63, *p* = 0.0032; Fig. 9*A*,*B*). Despite changes in OPC proliferation and abundance in the trisomic cortex, the percentage of CC1+/OLIG2+ mOLs was not significantly changed in the cortex of Ts65Dn mice (Fig. 9*C*). Likewise, we found no change in cortical *Mbp* mRNA levels through quantitative PCR (qPCR; Fig. 9*D*), or in MBP fluorescence intensity measured through immunostaining (Fig. 9*E*). Overall, this analysis suggests that OPC senescence alters progenitor abundance, but not OL maturation or myelination in the adult Ts65Dn cortex.

### Treatment with the senescence-reducing flavonoid fisetin rescues cellular and behavioral deficits in Ts65Dn mice

Senescent cell anti-apoptotic pathways (SCAPs) are a key feature of senescent cells, allowing them to evade apoptotic cell death (Kumari and Jat, 2021). SCAPs can be targeted by small molecule senolytic compounds to selectively clear senescent cells from pathologic tissues (Y. Zhu et al., 2015). Fisetin, a natural flavonoid, has senolytic properties and can be added to the diet as a supplement with minimal adverse effects (Elsallabi et al., 2022). We fed Ts65Dn mice a diet supplemented with 500-ppm fisetin, as previously described (Camell et al., 2021; Materials and Methods).

To begin investigating the cellular effects of fisetin treatment, we stained coronal brain sections from six-month mice for SA-β-gal activity under the same parameters as previously described. We observed a significant reduction in SA-β-gal positivity in both OLIG2+ (Genotype effect, *F*_(2)_ = 305.25, *p* = 2.5 × 10^−6^) and PDGFRA+ (Genotype effect, *F*_(4)_ = 24.87, *p* = 0.0011) cells in the cortex of six-month trisomic mice given fisetin (Fig. 8*B*,*C*). Fisetin supplementation also restored LMNB1 protein levels in Ts65Dn OPCs to CTL levels (Genotype effect, *F*_(6)_ = 15.86, *p* = 0.0059; Fig. 8*G*), increased the proportion of KI67+/PDGFRA+ cells in the Ts65Dn cortex (Genotype effect, *F*_(8)_ = 24.84, *p* = 0.0019; Fig. 8*H*), and restored the abundance of cortical OPCs (Genotype effect, *F*_(9)_ = 11.63, *p* = 0.017; Fig. 9*B*). While the number of OPCs was observed to significantly decrease in the three-month Ts65Dn CC, there was no significant difference in the number of OPCs in the CC at six months, and no effect of fisetin treatment on CC OPC cell counts. Likewise, there was a significant decrease in the number of KI67+/PDGFRA+ at both three months (*t*_(10)_ = 8.68, *p* = 0.013) and six months (Genotype effect, *F*_(11)_ = 6.73, *p* = 0.039), but no rescue of this reduction in proliferation activity through fisetin treatment at six months in the CC (Fig. 8*J*), consistent with a region-specific effect of senescent OPCs in the Ts65Dn cortex.

**Figure 10.**
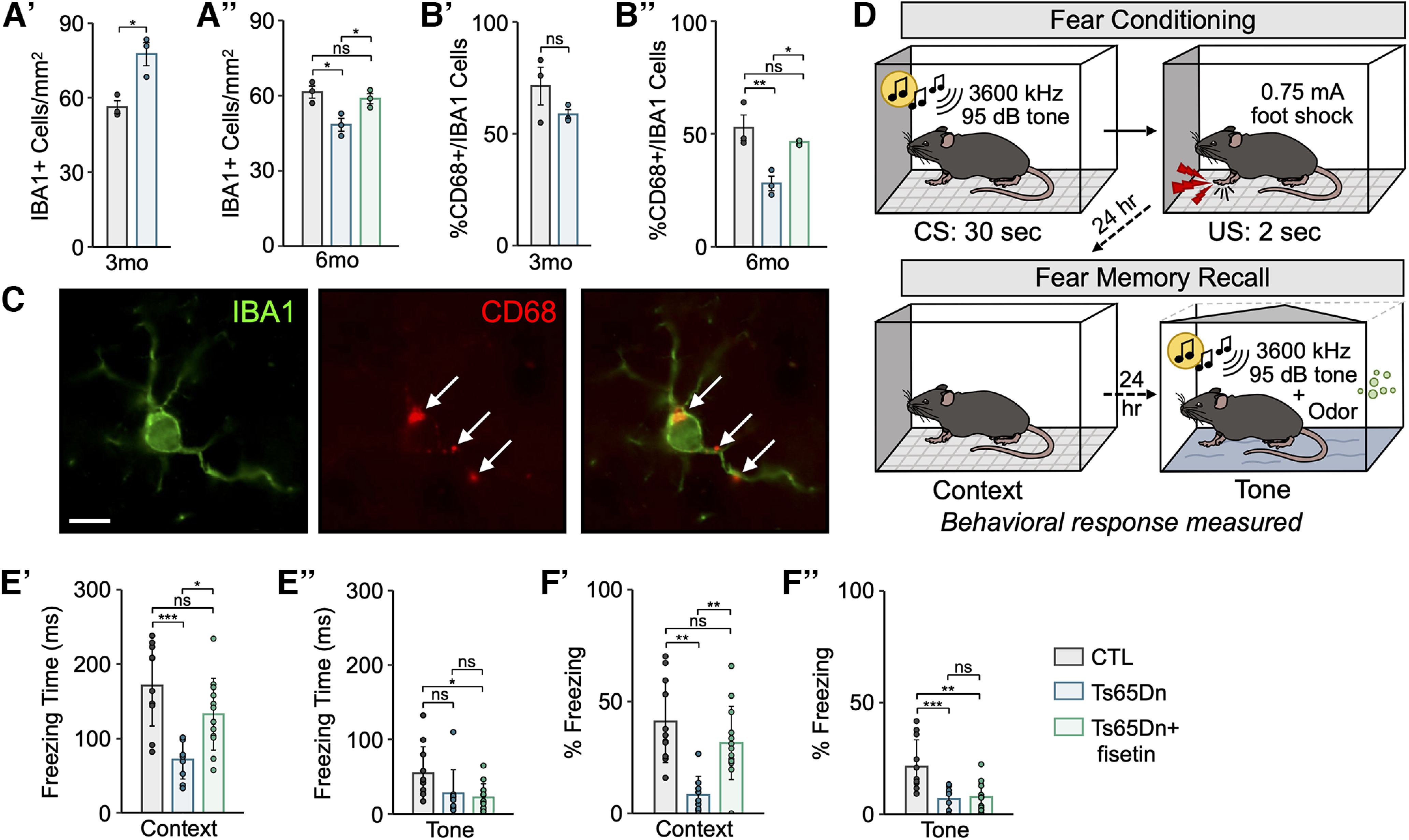
Fisetin rescues microglia phenotype and fear memory behavior in Ts65Dn mice. ***A’***, ***A’’***, Barplots of the number of IBA1+ (microglia) cells per mm^2^ in the cortex in three- and six-month mice, showing a significant increase in microglia in Ts65Dn at three months and a decrease at six months, with a rescue in six-month Ts65Dn mice treated with fisetin. Bars represent the average values for each condition (*n *=* *3 male mice per condition, *n *=* *3 replicates per mouse), and dots represent the average values for each mouse per condition. ***B’***, ***B’’***, as in ***A***, but depicting the percentage of CD68+/IBA1+ (activated microglia) in the cortex, showing a significant decrease in activated microglia at six months, with a rescue in six-month Ts65Dn mice treated with fisetin (*n *=* *3 male mice per condition, *n *=* *3 replicates per mouse). ***C***, Immunostaining for IBA1 (green) and CD68 (red) in a three-month Ts65Dn mouse. Images are representative of those observed in samples from Ts65Dn and CTL mice. Arrows point to CD68+ puncta in IBA1+ microglia. Scale bar is shown in the bottom left corner of the panel: 15 μm. ***D***, Schematic representation of fear conditioning and memory recall behavioral battery. ***E’***, ***E’’***, Barplots of the time to freezing behavior depending on context and tone, showing a significant decrease in context-based but not tone-based freezing in Ts65Dn mice, with a rescue in the context-based behavior in six-month Ts65Dn mice treated with fisetin. Bars represent the average values for each condition (*n *=* *9 male mice per condition), and dots represent the data collected for each mouse per condition. ***F’***, ***F’’***, As in ***E***, but depicting the percentage of freezing behavior, showing a significant decrease in context-based and tone-based freezing in Ts65Dn mice, with a rescue in the context-based, but not tone-based behavior in six-month Ts65Dn mice treated with fisetin (*n *=* *9 male mice per condition). For all plots: significance is determined using the two-tailed Student’s *t* test at three months, or using the one-way ANOVA and *post hoc* Tukey’s test at six months. *p-value < 0.05, **p-value < 0.01, ***p-value < 0.001, ns: not significant; error bars represent the average ± 1 standard deviation (SD).

DS mouse models demonstrate increased microglial and astrocyte reactivity, as well as increased inflammatory cytokine levels and interferon signaling (Jørgensen et al., 1990; Wilcock, 2012; C. Chen et al., 2014; Sullivan et al., 2016; Flores-Aguilar et al., 2020; Kong et al., 2020; Pinto et al., 2020; Ponroy Bally and Murai, 2021). Given that senescent cells modify the local inflammatory microenvironment through the SASP (Coppé et al., 2010), we hypothesized that fisetin administration and resulting reduction of senescent OPCs would alter local microglial phenotype. To begin, we counted the number of IBA1+ microglia in the cortex of three- and six-month Ts65Dn and CTL mice. We observed a significant increase in the number of microglia at three months and a decrease at the six-month time point (*t*_(12)_ = 4.43, *p* = 0.047; Genotype effect, *F*_(13)_ = 8.64, *p* = 0.018) in Ts65Dn mice relative to CTL. Fisetin administration was associated with a restoration of IBA1+ microglia in six-month trisomic mice (Genotype effect, *F*_(13)_ = 8.64, *p* = 0.046; Fig. 10*A*). Next, we tested whether the activation state of microglia was altered in Ts65Dn mice by quantifying the abundance of the phagocytic marker CD68 in IBA1+ cells. Interestingly, we observed a decrease in the proportion of CD68+/IBA1+ cells in Ts65Dn at six months (Genotype effect, *F*_(14)_ = 11.39, *p* = 0.0091; Fig. 10*B*,*C*), suggestive of a reduction in phagocytic microglial activity. This decline in microglial phagocytic capacity is consistent with prior reports demonstrating that aged microglia display a reduction or dysfunction in phagocytosis (Safaiyan et al., 2016; Marques et al., 2020; Thomas et al., 2022), thus suggesting that the Ts65Dn cortex and its associated senescent microenvironment may contribute to an impairment in microglia phagocytic activity, rescuable by fisetin treatment.

Individuals with DS, as well as mouse models of DS, exhibit pronounced learning and memory deficits that worsen with age. We thus wanted to determine whether senescence-reducing fisetin supplementation treatment could rescue learning-associated behavioral deficits in six-month trisomic mice. To do so, we employed a contextual fear conditioning paradigm, as previously done for Ts65Dn mice (P.J. Zhu et al., 2019), which pairs a foot shock (unconditioned stimulus, US) with a context (conditioned stimulus, CS), and subsequently tests memory strength by measuring fear-associated freezing behavior in mice exposed to the CS (Fig. 10*D* Materials and Methods). We found, as expected, that trisomic mice exhibited significantly lower freezing behavior compared with CTL littermates in both contextual and tone-associated freezing behavior (Genotype effect, *F*_(15)_ = 10.31, *p* = 0.0016; Genotype effect, *F*_(16)_ = 11.68, *p* = 0.00013; Fig. 10*E*,*F*). Contextual, but not tone-associated freezing behavior, was rescued in six-month trisomic mice treated with fisetin (Genotype effect, *F*_(15)_ = 10.31, *p* = 0.0011), indicating improved associative learning (Fig. 10*E*,*F*). Overall, the behavioral data appears to suggest that, in Ts65Dn mice, cognitive impairment may be caused, at least in part, by cortical OPC senescence and that this behavioral impairment is rescuable by fisetin treatment.

## Discussion

The present study leverages an integrated transcriptome and chromatin accessibility landscape of the mature Ts65Dn mouse cortex to identify trisomic OPCs that undergo accelerated senescence. The study characterizes cell type-specific effects of trisomy and dosage compensation, identifying putative mechanisms of cell-cell signaling dysfunction in the mature brain. Importantly, we assemble multiple lines of evidence supporting OPC senescence in Ts65Dn mice, and we find that OPC senescence is spatially concentrated in deep cortical layers. This suggests a unique phenotype for cortical gray matter OPCs and a role for the cortical niche in programming OPC state, similar to what has been recently reported for other non-neuronal cells (Stogsdill et al., 2022).

Our work builds on previous data suggesting cell-autonomous defects in OL maturation in mouse models of DS, as our data confirms a reduction in OPC proliferation and fewer mOLs in the CC of Ts65Dn mice. In the developing DS brain, there is a cell fate shift from neurogenesis to gliogenesis, resulting in a transient increase in OLIG2+ cells, followed by a decrease in mOLs and an increase in astrocytes with age, which is thought, in part, to be driven by triplication of the OLIG1/2 TFs (Reiche et al., 2019). This supports a model of defective OL-lineage differentiation capacity and a shift in glial cell commitment. Importantly, our data show that at six months, Ts65Dn mice show a reduction in cortical gray matter OPCs, but not CC OPCs, suggesting an age-restricted cortical gray matter phenotype. Therefore, trisomic OPCs are uniquely vulnerable to impaired maturation, but the local cortical microenvironment may be important as a second hit to promote accelerated senescence.

There is growing recognition that OPCs are an important mediator of neuroinflammation and aging phenotypes in the brain. OPCs exhibit regional, transcriptional, and functional heterogeneity, including differences in calcium signaling and electrical activity (Falcão et al., 2018; Marisca et al., 2020). OPCs become more regionally diverse, acquire ion channels, and undergo an inflammatory transformation with age (Neumann et al., 2021). OPCs harbor a highly dynamic cytokine receptor-mediated surveillance mechanism that responds to cues of injury and inflammation (Kirby et al., 2019), and these receptors promote neuroinflammatory responses on IFN signaling. OPC signaling to the microenvironment is bidirectional: local inflammation influences OPC ability to proliferate and differentiate, while OPCs also exert an immunomodulatory function on neighboring cells. Therefore, our study adds to this existing literature to suggest a region-specific disruption in OPC function with aging in DS.

The DS brain exhibits several pathologic changes, including DNA damage, chromatin instability, oxidative stress, mitochondrial dysfunction, and immune activation, that may predispose to cellular senescence. In particular, there is a chronic hyper-interferon state in the DS brain because of the increased gene dosage of IFNARs (Sullivan et al., 2016; Araya et al., 2019; Waugh et al., 2019; M. Jin et al., 2022). IFNAR inhibition reverses senescent microglia phenotype in human DS cells, and chronic interferon-opathy may contribute to accelerated aging and neurodegeneration in DS (M. Jin et al., 2022). Nevertheless, the precise gene-environment mechanisms that regulate region-specific OPC senescence, at least in Ts65Dn mice, are unclear. Interestingly, our results are consistent with previous studies of the aged AD-affected brain, in which inflammatory Aβ plaque-associated OPCs were found to exhibit increased senescence (P. Zhang et al., 2019). This suggests a link between OPC senescence and a neuroinflammatory microenvironment in aging. Other investigators have reported senescence in human DS neural progenitor cells (NPCs) and microglia, but these studies were done in human cell culture models (Meharena et al., 2022). Our findings are exclusively derived from the Ts65Dn mouse model, and this does not exclude neural progenitor or microglia senescence at other developmental or stimulus-dependent contexts. Further validation of these findings in human tissue across the lifespan will be critical to understanding the role of senescence in individuals with DS.

There are several limitations to the present study. Droplet-based single-cell genomics is limited by gene and chromatin fragment “drop-out” and is sparser than bulk approaches. snRNA-seq is biased against lowly expressed genes, which includes many immune molecules and senescence-associated transcripts in the brain. Chromatin accessibility may be a coarse indicator of senescence-associated epigenetic changes in OPCs, and a detailed analysis of the histone landscape and 3D genome architecture of trisomic OPCs is warranted. Furthermore, gene expression levels may not correlate with protein levels, particularly with aging, and our data do not reflect possible post-transcriptional regulatory mechanisms in translational control. The use of the Ts65Dn mouse model also harbors inherent limitations, namely the inclusion of only ∼55% of Hsa21 protein-coding orthologs (Escorihuela et al., 1995). Indeed, Ts65Dn mice do not triplicate many of the trisomic human loci, do not exhibit β-amyloid plaques consistent with human AD (Choi et al., 2009), and create trisomic imbalance for several Mmu17 genes unrelated to Hsa21. A recent study conducted on a newly-developed DS model, Ts66Yah, demonstrates that several genes located on Mmu17 that are triplicated in Ts65Dn mice may play a role in DS-associated neuropathology and behavior (Duchon et al., 2022). Conclusions drawn from use of the Ts65Dn mouse must therefore be interpreted and understood in the context of existing limitations of the model, and warrant supplementation in other murine contexts or in human DS tissue for increased validity and translatability. Further, senescence-reducing therapy with fisetin is not specifically targeted to OPCs, and the effects of fisetin may be related to noncell-autonomous mechanisms. This does not exclude the possibility of direct effect of fisetin on other cell types. Fisetin also exhibits anti-inflammatory, neuroprotective, and antioxidant activity (L.T. Zheng et al., 2008; Khan et al., 2013; Huang et al., 2018; Molagoda et al., 2021), and has recently been shown to reduce mortality in aged virally-infected mice (Camell et al., 2021). It is therefore possible that fisetin administration could exert a beneficial impact on disease pathogenesis, gliosis, and cognitive function via mechanisms that do not directly involve senescence reduction. Future work using transgenic models to selectively deplete senescent OPCs will be important to demonstrate the importance of this phenomenon to DS-associated cognitive decline and neurodegeneration.

Despite these limitations, our study identifies a cell type-specific senescence phenotype in Ts65Dn OPCs that is targetable with senescence-reducing therapies. These findings shed light on potential drivers of chronic inflammation and cognitive decline in the DS brain. Furthermore, the integrated single-nucleus transcriptomic and chromatin landscape in the Ts65Dn brain is an important resource for discovery in DS neurobiology.

## Synthesis

Reviewing Editor: Benjamin Deneen, Baylor College of Medicine Decisions are customarily a result of the Reviewing Editor and the peer reviewers coming together and discussing their recommendations until a consensus is reached. When revisions are invited, a fact-based synthesis statement explaining their decision and outlining what is needed to prepare a revision will be listed below. The following reviewer(s) agreed to reveal their identity: John Lukens, Yungki Park.

The reviewers found this paper to be of interest as a multiomics analysis of mouse models of Down’s could be a useful resource in the field. Despite these strengths Reviewer 2 found some shortcomings with the bioinformatics analysis and some of the statistical approaches that were employed. We feel strongly that these issues need to be resolved before this paper can be published. Also, reviewer 1 suggested highlighting some of the limitations of the study, which we also agree with. Please proceed with an appropriate revision, incorporating these comments.
