# Supplementary figures and images for: Single-Nucleus Profiling Identifies Accelerated Oligodendrocyte Precursor Cell Senescence in a Mouse Model of Down Syndrome
Source: eNeuro. 2023 Aug 14;10(8):ENEURO.0147-23.2023. doi: 10.1523/ENEURO.0147-23.2023 (PMC10449487; doi:10.1523/ENEURO.0147-23.2023)

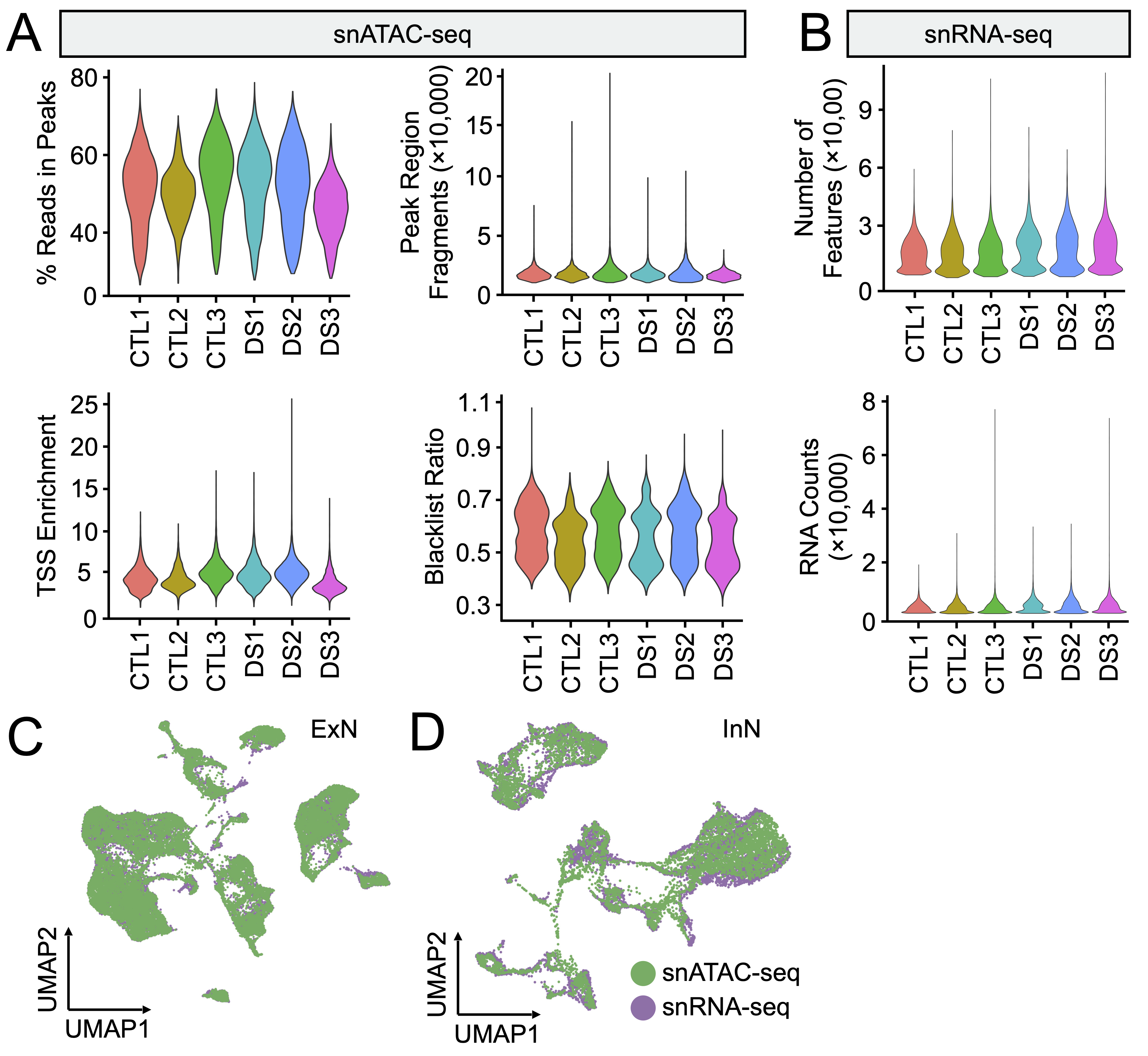

Supplement: Extended Data Figure 1-1 — Quality control (QC) metrics for multiomic single-nucleus data and integration of snATAC-seq and snRNA-seq data for neuronal subsets, related to Figure 1. A, Quality control (QC) metrics for snATAC-seq data, including the percentage of reads in peaks, the number of peak region fragments, transcription start site (TSS) enrichment, blacklist ratio, and nucleosome signal per replicate from six-month Ts65Dn and euploid control (CTL) mice (n = 3 male mice per condition). B, As in A, but depicting QC metrics for snRNA-seq data, including the number of features, total transcript counts, and percent mitochondrial content per replicate. C, UMAP visualization of multiomic integration of snATAC-seq and snRNA-seq datasets colored by originating dataset for the excitatory neuron (ExN) subset. D, As in C, but depicting multiomic integration for the inhibitory neuron (InN) subset. Download Figure 1-1, TIF file. [file enu-eN-NWR-0147-23-s02.tif]
